# NBTXR3 improves the efficacy of immunoradiotherapy combining nonfucosylated anti-CTLA4 in an anti-PD1 resistant lung cancer model

**DOI:** 10.3389/fimmu.2022.1022011

**Published:** 2022-11-03

**Authors:** Yun Hu, Sébastien Paris, Genevieve Bertolet, Hampartsoum B. Barsoumian, Qi Wang, Jordan Da Silva, Nalini B. Patel, Nguyen Nguyen, Denaha J. Doss, Ailing Huang, Ethan Hsu, Claudia S. Kettlun Leyton, Tiffany A. Voss, Fatemeh Masrorpour, Carola Leuschner, Jordan T. Pietz, Nahum Puebla-Osorio, Saumil Gandhi, Quynh-Nhu Nguyen, Jing Wang, Maria Angelica Cortez, James W. Welsh

**Affiliations:** ^1^ Department of Radiation Oncology, The University of Texas MD Anderson Cancer Center, Houston, TX, United States; ^2^ Department of Translational Science, Nanobiotix, Paris, France; ^3^ Department of Bioinformatics and Computational Biology, The University of Texas MD Anderson Cancer Center, Houston, TX, United States; ^4^ Department of Leukemia, The University of Texas MD Anderson Cancer Center, Houston, TX, United States; ^5^ Department of Genetics, The University of Texas MD Anderson Cancer Center, Houston, TX, United States; ^6^ Department of Strategic Communication, The University of Texas MD Anderson Cancer Center, Houston, TX, United States

**Keywords:** immunoradiotherapy, NBTXR3 nanoparticle, immume checkpoint blockade, abscopal effect, lung cancer

## Abstract

The efficacy of immunoradiotherapy consisting of radiation therapy and immune checkpoint blockade relies on effectively promoting the systemic antitumor immune response’s activation while simultaneously reducing local factors favoring immune suppression. We previously demonstrated that NBTXR3, a nanoparticle radioenhancer, significantly improved immune responses in a murine anti-PD1-resistant metastatic lung cancer model. We hypothesize that radioactivated-NBTXR3 addition to anti-PD1 and a second-generation anti-CTLA4 could improve treatment effectiveness. To test this hypothesis, we inoculated mice with 344SQR cells in the right and left legs to establish primary and secondary tumors. The primary tumors were intratumorally injected with NBTXR3 nanoparticles on day 7, followed by three fractions of 12 Gy radiation on days 8, 9, and 10. The secondary tumors received two fractions of 1Gy radiation on days 13 and 14. Multiple rounds of anti-PD1, anti-CTLA4 or nonfucosylated anti-CTLA4 were given to the mice. Immune profiling of the tumors revealed that the combination of NBTXR3 with immunoradiotherapy significantly upregulated the activities of a wide range of antitumor immune pathways and reduced the abundance of regulatory suppressor T cells. This combination effectively eradicated the primary and secondary tumors and increased animal survival to 75%. Remarkably, previously treated with NBTXR3-containing treatment, the survivor mice exhibited a long-lasting antitumor memory immune response. This data provides compelling evidence of the efficacy of NBTXR3 to synergize with the immunoradiotherapy approach when combined with an anti-PD1 and multiple checkpoints such as a second generation anti-CTLA4 and show the potential for clinical uses of antitumor immunomodulatory effects of NBTXR3.

## Graphical Abstract

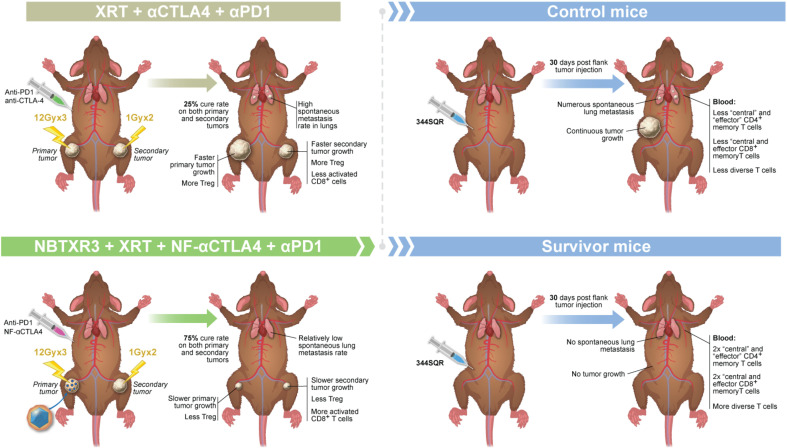

## Introduction

Checkpoint inhibitors (CPIs) have been a revolution in cancer therapy (1), boasting unprecedented responses in many cancers deemed previously intractable (2). The first CPI was directed against cytotoxic T lymphocyte antigen 4 (CTLA4) (3, 4). CTLA4 is a competitive antagonist with the T cell co-receptor CD28, which delivers the second signal essential for full T cell activation. By binding to the same ligands as CD28, CTLA4 denies the T cell this activating signal, replacing it with an inhibitory one (5). Anti-CTLA4 (αCTLA4) binds to the extracellular region of CTLA4, preventing it from binding the B7 ligands and thereby blocking this inhibitory signal (6).

Since its approval in 2011, αCTLA4 has become a mainstay of cancer immunotherapy (7). Both as a monotherapy and in combination with αPD1, αCTLA4 has been widely used in many types of solid tumors with significant treatment benefits (8). Preclinical results demonstrated that a nonfucosylated (NF) version of αCTLA4 (NF-αCTLA4) may achieve better treatment outcomes than traditional αCTLA4 by increasing Treg depletion at the tumor site (9). The lack of a fucosyl group on the fragment crystallizable (Fc) region of the NF-αCTLA4 antibody results in this region being bound with higher affinity by the Fcγ receptor CD16, which is predominantly expressed on natural killer (NK) cells. The binding of antibodies by CD16 triggers antibody-dependent cellular cytotoxicity (ADCC), resulting in the death of any cells expressing CTLA4. As CTLA4 is predominately expressed by T regulatory cells (Tregs), this results in the preferential depletion of this immunosuppressive cell population (10, 11). Thus, NF-αCTLA4 improves upon existing αCTLA4 treatments by effectively reducing the tumor-resident Treg population (12).

More recently, immunoradiotherapy that combines stereotactic body radiation therapy (SBRT) with CPIs (both αCTLA4 and αPD1) has proven effective in treating metastatic cancers by inducing systemic and specific antitumor immune responses (13–15). To maximize the efficacy, it is crucial to promote the priming of effector cells and reduce concomitant immune suppression. NBTXR3, a hafnium oxide nanoparticle, was initially introduced as a radiation-enhancer for treating localized tumors (16). Lately, it has been discovered that NBTXR3-mediated radiotherapy can also serve as an immune enhancer that promotes antitumor activities, extending its treatment benefits to distant lesions (17–19). In preclinical models, NBTXR3 was found to facilitate the infiltration of CD8^+^ T cells into abscopal tumors and elevate the expression of genes that favor tumor killing. Phase I clinical data evaluating NBTXR3/RT/αPD1 in patients with advanced cancers show that intratumoral injection of NBTXR3 is feasible and well-tolerated with promising signs of efficacy (20). These findings are of great significance, as only a minority of cancer patients (<20%) respond to αPD1 treatment (21). Given the excellent treatment potential of NBTXR3 and NF-αCTLA4, we hypothesize that a novel immunoradiotherapy integrating NBTXR3, radiotherapy, NF-αCTLA4, and αPD1 would further improve the treatment outcome of αPD1-resistant lung cancer.

## Materials and methods

### Materials

NBTXR3 nanoparticles were kindly provided by Nanobiotix and were stored at room temperature in darkness before use. Bristol-Myers Squibb kindly provided the mouse αCTLA4, NF-αCTLA4, and αPD1 antibodies. Antibodies for flow cytometry, including αCD45:PE-Cy7 (Cat. #147704), αCD4:APC-Cy7 (Cat. #100414), αCD8:PerCP-Cy5.5 (Cat. #126610), αCD3:BV510 (Cat. #100234), αCD49b:APC (Cat. #108910), αCD19:PE/Dazzle (Cat. #115554), αFoxP3:PE (Cat. #126404), αGranzyme B:Pacific Blue (Cat. #372218), αCD45:Pacific Blue (Cat. #103126), αCD4:APC/Fire750 (Cat. #100460), αCD44:APC (Cat. #103012), αCD62L:PE-Cy7 (Cat. #104418), and αCD27:AF700 (Cat. #124240) were purchased from BioLegend.

### Cell line and culture conditions

The cell line used throughout this study was αPD1-resistant lung cancer cell line 344SQR (22). It was cultured by methods described in previous reports (17, 19).

### Tumor establishment and treatment

The animals used in all the experiments in this study were 8-12-week-old 129/SvEv female mice purchased from Taconic Biosciences. The 344SQR cells (5×10^4^ in 100 µL PBS) were injected into the right leg on day 0 to create primary tumors and into the left leg on day 4 to create secondary tumors. Tumor size was monitored with digital calipers at least twice a week, and tumor volumes were calculated using the formula: tumor volume = 0.5 × width^2^ × length. Mice were divided into six treatment groups, with eight mice in each group: 1) control (no treatment), 2) NBTXR3+XRT, 3) XRT+αCTLA4+αPD1, 4) XRT+NF-αCTLA4+αPD1, 5) NBTXRT3+XRT+αCTLA4+αPD1, and 6) NBTXRT3+XRT+NF-αCTLA4+αPD1. NBTXR3 nanoparticles in 5% glucose with 25% of the tumor volume were intratumorally injected into the primary tumors on day 7. CPIs, including αCTLA4 (50 μg), NF-αCTLA4 (50 μg), and αPD1 (200 μg), were intraperitoneally injected into mice on days 7, 11, and 14. For experiments that evaluate the efficacy of intratumoral injection of NF-αCTLA4, the primary tumors were intratumorally delivered with 50 μg NF-αCTLA4 on day 7 (IT1) or on days 7 and 11 (IT2). Anti-PD1 treatment continued on days 21, 28, 35, and 42. The primary tumors were irradiated with three fractions of 12 Gy, each with a PXi X-Rad SmART irradiator on days 8, 9, and 10 (total dose of 36 Gy). The secondary tumors were irradiated with two fractions of 1 Gy each, also with a PXi X-Rad SmART irradiator on days 13 and 14 (total dose of 2 Gy). The dose was delivered with two opposing beams from anteroposterior and posteroanterior positions and a 15-mm circular collimator. The dosimetry and treatment planning was performed using the Advanced Treatment Planning software supplied by the vendor. Precision XRay Corporation commissioned all collimators at the time of installation. Routine output checks were done with an ion chamber to ensure that the outputs had not changed and that the treatment plans were accurate. Mice were euthanized when primary or secondary tumors reached 14 mm in any dimension. All animal procedures followed the guidelines of the Institutional Animal Care and Use Committee at The University of Texas MD Anderson Cancer Center.

### Tumor rechallenge

Mice from the XRT+NF-αCTLA4+αPD1, NBTXR3+XRT+αCTLA4+αPD1, and NBTXR3+XRT+NF-αCTLA4+αPD1 groups that had survived more than 156 days past the initial tumor challenge were rechallenged with 5×10^4^ 344SQR cells in 100 μL PBS in their right flank. Five mice of similar age were also implanted with the same number of 344SQR cells and served as control. No further treatment was given. As before, mice were euthanized when the tumor reached 14 mm in cross-section. The blood samples were collected 20 days before tumor rechallenge and 7 and 21 days post tumor rechallenge for immune profiling. The lungs were also harvested at the end of the experiment to count the number of lung metastases.

### Tumor processing

Primary and secondary tumors were harvested on day 16 for flow cytometric immune profiling and on day 18 for NanoString analysis. The tumor tissues were minced and digested with 250 µg/mL of Liberase (Roche, Cat. #05401127001) and 20 µg/mL DNAse (Sigma-Aldrich, Cat. #4716728001) at 37°C for 30 min. The digestion process was stopped with 1 mL fetal bovine serum (FBS), and the samples were filtered and washed with PBS (2% FBS) buffer. The cells were either stained with antibodies for flow cytometry analysis (FACS) or frozen in TRIzol for RNA extraction.

### Flow cytometric analysis

The above-processed cells on day 16 were stained with αCD45:PE-Cy7, αCD4:APC-Cy7, αCD8:PerCP-Cy5.5, αCD3:BV510, αCD49b:APC, αCD19:PE/Dazzle, αFoxP3:PE, and αGranzyme B:Pacific Blue. The blood samples from the tumor rechallenge study were collected on day 21 post tumor rechallenge and were stained with αCD45:Pacific Blue, αCD4:APC/Fire 750, αCD8:PerCP-Cy5.5, αCD44:APC, αCD62L:PE-Cy7, αCD3:BV510, αCD19:PE/Dazzle, and αCD27:AF700. Samples were analyzed using a Gallios Flow Cytometer (Beckman Coulter) with the Kaluza software Version 2.1.

### Counting numbers of lung metastases

The lungs collected either on day 16 or at the end of the tumor rechallenge experiment were stored in Bouin’s fixative solution (Polysciences Inc., Cat. #16045-1) for 3 days, after which the number of lung metastases was counted.

### Analysis of immune-related genes in tumor immune microenvironment *via* Nanostring

Total RNA extracted from both primary and secondary tumors harvested on day 18 were analyzed with an nCounter PanCancer Immune Profiling Panel and an nCounter MAX Analysis System (both from NanoString Technologies, Seattle, WA, USA) by following the manufacturer’s instructions; the expression of immune-related genes was analyzed with the PanCancer Immune Profiling Advanced Analysis Module (also from NanoString Technologies).

### TCR repertoire analysis

Blood was collected from 4 mice in the control group and the NBTXR3+XRT+NF-αCTLA4+αPD1 group 21 days post tumor rechallenge. Total RNA was subsequently extracted from the blood. TCR analysis was performed using a method described in the previous study (17). The raw TCR sequencing data was processed using MiXCR (version 3.0.13) with default parameters (23). In brief, the raw reads were aligned to the *Mus musculus* reference T cell receptor genes based on the ImMunoGeneTics database (IMGT) (24). Then, the aligned reads were assembled to construct the CDR3 (complementarity-determining region 3). Finally, the MiXCR reported the clonotypes of each sample, which the unique clonotype was defined as the unique CDR3 amino acid sequences and V-J segments genes. Further bioinformatics analysis of the TCR beta chain and data visualization was performed using the Immunarch package in R (version 4.0.1) (25). The circlize package was used to generate the circos plot of each sample regarding V-J usage (26).

### Statistical analyses

All statistical analyses were performed with Prism 9.0.0 (GraphPad Software). Tumor volumes were compared by two-way ANOVA and were expressed as mean tumor volume ± standard error of the mean (SEM). Mouse survival rates were compared with the Kaplan–Meier method and log-rank tests. NanoString data were compared by one-way ANOVA or two-tailed t tests. All other data were compared with two-tailed t tests and expressed as mean value ± SEM. P values of < 0.05 were considered to indicate statistically significant differences.

## Results

### The combination of NBTXR3 with NF-αCTLA4 and αPD1 immunoradiotherapy leads to improved tumor control and increased survival rates

**Figure 1:**
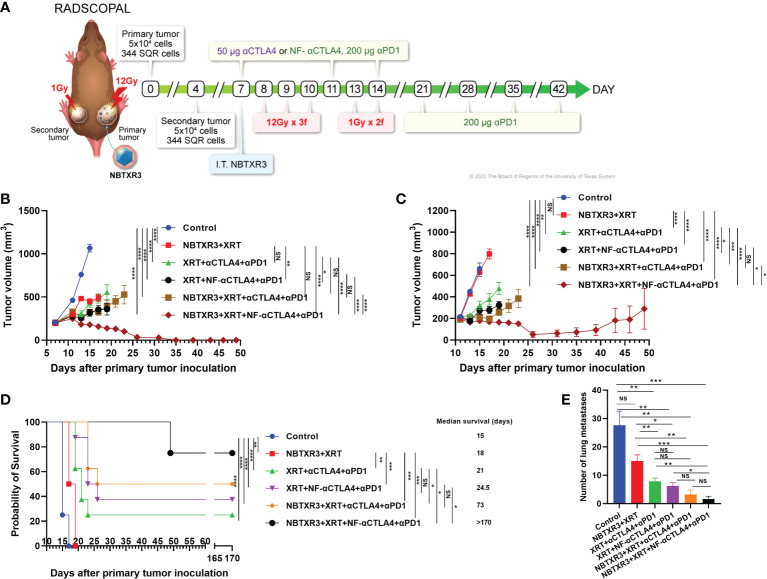
NF-αCTLA4, combined with NBTXR3-mediated immunoradiotherapy, significantly improves treatment efficacy in αPD1-resistant lung cancer. **(A)** Treatment schema for combination therapies of NBTXR3, XRT, αCTLA4 or NF-αCTLA4, and αPD1. Mice were subcutaneously injected with 5 × 10^4^ 344SQR cells in the right legs on day 0 (to establish primary tumors, to be irradiated with high dose radiation) and in the left legs on day 4 (to establish secondary tumors, to be irradiated with low dose radiation). NBTXR3 was delivered to the primary tumor by intratumoral injection on day 7. Primary tumors were treated with three 12 Gy fractions on days 8, 9, and 10. Secondary tumors were irradiated with two 1 Gy fractions on days 13 and 14. αPD1 (200 μg) and anti-CTLA4 or NF-αCTLA4 (50 μg) were given by intraperitoneal injection on days 7, 11, and 14. αPD1 treatment was continued once a week from day 21 until day 42. For intratumoral injections of NF-αCTLA4, 50 μg of NF-αCTLA4 was intratumorally injected into the primary tumors on days 7 and 11. **(B)** Tumor volumes of the primary tumor. **(C)** Tumor volumes of the secondary tumor. **(D)** Survival rates and median survival time. **(E)** The number of spontaneous lung metastasis on day 16. Data are expressed as means ± SEM. P< 0.05 was considered statistically significant. *P< 0.05, **P< 0.01, ***P< 0.001, ****P< 0.0001, NS, not significant.

Mice were challenged with a two-tumor system, as shown in **Figure 1A**, to simulate a primary tumor and a secondary metastatic site. Mice were then treated with XRT supplemented with the NBTXR3 nanoparticle. Although this treatment was effective at restraining the growth of the primary tumor (**Figure 1B**), it did not affect the secondary tumors (**Figure 1C**) and, consequently, only a limited (albeit statistically significant) effect on median survival (+3 days; **Figure 1D**).

We began by comparing the ability of αCTLA4 vs. NF-αCTLA4 to augment immunoradiotherapeutic control of tumor growth. Mice were next treated with XRT+αPD1 and either αCTLA4 or NF-αCTLA4. XRT+αCTLA4+αPD1 was no better than XRT+NBTXR3 at limiting primary tumor growth (**Figure 1B**), although it was significantly better at slowing secondary tumor growth (**Figure 1C**), conferring a significantly improved median survival and an overall survival rate of 25% (2/8 mice; **Figure 1D**). However, substituting NF-αCTLA4 for αCTLA4 produced better results still; not only control of the primary tumor was greater than either NBTXR3+XRT or XRT+αCTLA4+αPD1 (**Figure 1B**), but the growth of the secondary tumor was also significantly slower (**Figure 1C**), and overall survival was increased to 37.5% (3/8 mice; **Figure 1D**) (although the difference in median survival between the XRT+αCTLA4+αPD1 and the XRT+NF-αCTLA4+αPD1 groups was not significant).

We further amplified these two radioimmunological combinations with the NBTXR3 nanoparticle. The addition of NBTXR3 to XRT+αCTLA4+αPD1 improved primary and secondary tumor control to a level matching that achieved by XRT+NF-αCTLA4+αPD1 (**Figures 1B, C**) and achieved an overall survival rate of 50% (4/8 mice; **Figure 1D**), with a significantly longer median survival than XRT+αCTLA4+αPD1. Nevertheless, the most potent combination of all was that of NBTXR3+XRT+NF-αCTLA4+αPD1. This combination, and this combination alone, achieved 100% complete remission of the primary tumors (8/8 mice; **Figure 1B**, **Supplementary Figure 1**) and 75% complete remission of the secondary tumors (6/8 mice; **Figure 1C**, **Supplementary Figure 1A**). Both overall and median survival was highest in this group, with 75% overall survival and a median survival of >170 days. Thus, the treatment groups displayed an increasing level of tumor control, with NBTXR3 amplifying the local effects of radiation, CPI in the form of αCTLA4 and αPD1 boosting the systemic immune response against the tumors, and the substitution of αCTLA4 with NF-αCTLA4 boosting this even further. This was reflected when we examined the mice’s lungs for the presence of metastatic nodules; we found that the number of metastases was reduced roughly in proportion to the relative degree to which the primary and secondary tumors were controlled by each treatment group (**Figure 1E**). In addition, the treatment efficacy of intratumoral injection of NF-αCTLA4 was evaluated. As shown in **Supplementary Figure 1B**, intratumoral injection of NF-αCTLA4 and various combinations of XRT, NBTXR3, and αPD1 delayed the growth of both tumors and survival compared to the control group. Remarkably, compared to two times injection of NF-αCTLA4 without NBTXR3 (XRT+NF-αCTLA4(IT2)+αPD1), single time intratumoral injection of NF-αCTLA4 with NBTXR3 (NBTXR3+XRT+NF-αCTLA4(IT1)+αPD1) achieved similar primary tumor control, and significantly improved secondary tumor control and increased the survival rate from12.5% to 28.5%. Interestingly, two times injection of NF-αCTLA4 with NBTXR3 (NBTXR3+XRT+NF-αCTLA4(IT2)+αPD1 did not improve tumor control compared to (NBTXR3+XRT+NF-αCTLA4(IT1)+αPD1 but starkly increased the percentage of cured mice (28.5% to 57.1%). Remarkably, the percentage of cured mice achieved by XRT+NF-αCTLA4(IT2)+αPD1 (12.5%) was multiplied by 4.57 times when NBTXR3 was added to this combination (NBTXR3+XRT+NF-αCTLA4(IT2)+αPD1.

### NBTXR3, in combination with NF-αCTLA4, reduces Tregs and activates CD8^+^ T cells in the tumor immune microenvironment

**Figure 2:**
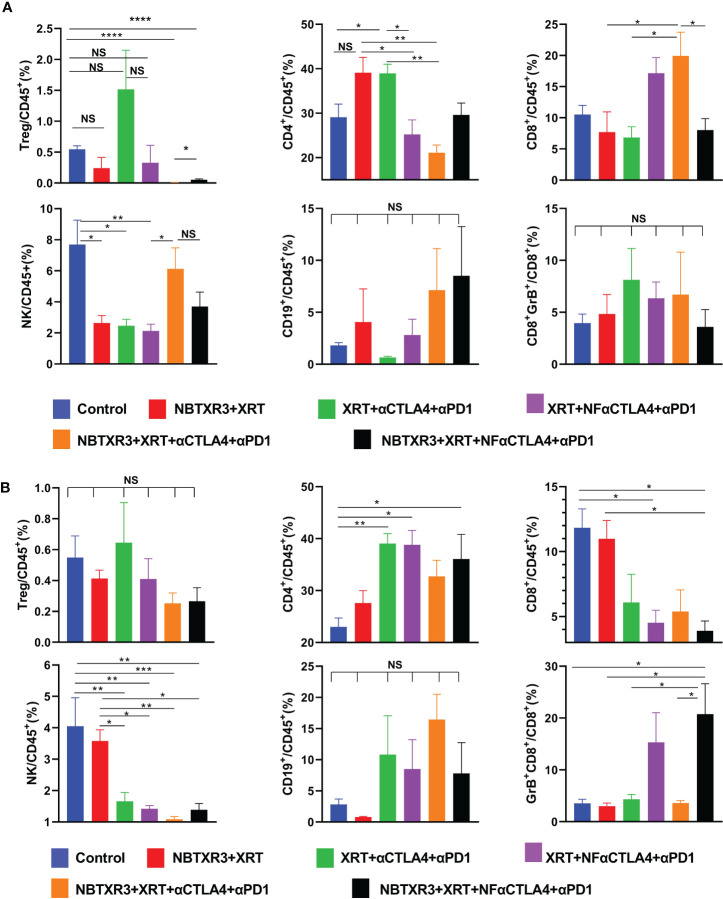
NF-αCTLA4 and NBTXR3-mediated immunoradiotherapy significantly reduces Tregs and promotes CD8^+^ T cell activation in the tumor immune microenvironment. **(A)** Percentages of various immune cells in the primary tumor. **(B)** Percentages of various immune cells in the secondary tumors. The mice (n=5) were treated with various combination therapies as indicated in **Figure 1A**, and both primary and secondary tumors were harvested on day 16. Immune cell populations, including CD3^+^CD4^+^FoxP3^+^(Tregs), CD3^+^CD4^+^ T cells, CD3^+^CD8^+^ T cells, CD3^-^CD49b^+^ (NK cells), CD3^-^CD19^+^ (B cells) and the Granzyme B^+^ (Gzm B^+^) CD3^+^CD8^+^ T cells subpopulation, were analyzed by flow cytometry. Data are expressed as means ± SEM. P< 0.05 was considered statistically significant. *P< 0.05, **P< 0.01, ***P< 0.001, ****P< 0.0001, NS, not significant.

Next, we used flow cytometry to explore how each combination therapy affected the tumor immune microenvironment of both primary and secondary tumors. As shown in **Figure 2**, radiotherapy consisting of NBTXR3+XRT without CPI produced no significant alterations in any measured cell population in the primary or secondary tumor, other than a reduction in NK cells in the primary tumor. However, a trend towards a decrease in Tregs and an increase in the CD4^+^ population can be observed in both tumors. XRT, combined with CPI (consisting of αCTLA4+αPD1), also reduced NK cells in the primary tumor. In addition, treatment with XRT+αCTLA4+αPD1 resulted in a significant increase in the percentage of CD4^+^ T cells (as well as a trend to Treg increase) and a concomitant decrease in the percentage of CD8^+^ T cells and NK cells within the secondary tumor. Substitution of NF-αCTLA4 for αCTLA4 significantly reduced the percentage of CD4^+^/CD45^+^ and increased CD8^+^/CD45^+^ in the primary tumor. On the secondary, substituting NF-αCTLA4 for αCTLA4 significantly increases Gzm B^+^ CD8^+^/CD8^+^ T cells.

Interestingly, adding NBTXR3 to either the XRT+αCTLA4+αPD1 group or XRT+NF-αCTLA4+αPD1 group led to an almost complete absence of Tregs in the primary tumor as compared to the control group. In the NBTXR3+XRT+αCTLA4+αPD1 group, this came at the cost of significantly reducing overall CD4^+^ T cells (as compared to the levels measured in the XRT+αCTLA4+αPD1 group). The NBTXR3+XRT+NF-αCTLA4+αPD1 group, on the other hand, achieved a reduction in Tregs equal to that of the NBTXR3+XRT+αCTLA4+αPD1 group without this cost to the CD4^+^/CD45^+^ overall population. Moreover, the secondary tumors of the NBTXR3+XRT+NF-αCTLA4+αPD1 group boasted the lowest average Treg levels while also displaying significantly higher overall CD4^+^ levels. This was accompanied by the highest percentage of granzyme B^+^ CD8^+^ T cells in the secondary tumors of any group. Overall, the use of NF-αCTLA4 and NBTXR3 reduced Tregs in both primary and secondary tumors, and both in tandem did so while preserving CD4^+^ T cell levels and increasing cytotoxic T cell levels in the secondary tumor. The quadruple therapies did not result in significant changes in B cell populations relative to the control.

### NBTXR3, in combination with NF-αCTLA4, modulates immune-related gene expression that favors antitumor activity

**Figure 3:**
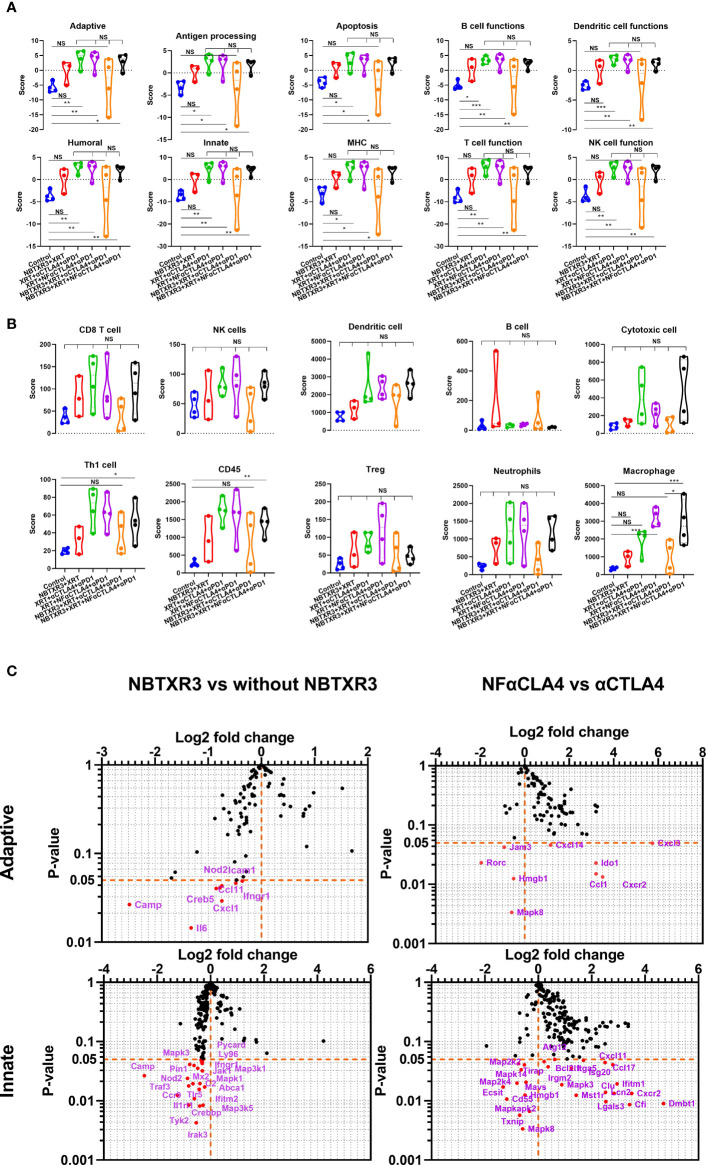
NBTXR3, combined with NF-αCTLA4, modulates immune-related gene expression to improve the primary tumor’s antitumor immune response. **(A)** Activities of immune pathways in the primary tumors. **(B)** The score of immune cell abundance in the primary tumor. **(C)** Changes in gene expression in adaptive and innate immune pathways. Mice bearing 344SQR tumors were treated with various immunoradiotherapies as indicated in Figure 1A, and the primary tumors were harvested on day 18. The RNA extracted from the tumors was analyzed by a nCounter PanCancer Immune Profiling Panel. Data are expressed as means ± SEM. P< 0.05 was considered statistically significant. *P< 0.05, **P< 0.01, ***P< 0.001, NS, not significant.

To better understand how these combination therapies affected immune activities at the genetic level, RNA was isolated from the primary and the secondary tumors harvested on day 18. This RNA was analyzed using the NanoString PanCancer Immune Profiling Panel. As shown in **Figure 3A**, NBTXR3+XRT tends to enhance immune pathways in the primary tumors, but only B cell function has significantly increased. However, combination therapies, including XRT+αCTLA4+αPD1, XRT+NF-αCTLA4+αPD1, NBTXR3+ XRT+NF-αCTLA4+αPD1 involving CPI succeeded in promoting the activities of various immune pathways, such as the adaptive pathway, antigen processing, B cell function, T cell function, NK cell function, *etc.* Whether CTLA4 blockade was mediated by NF-αCTLA4 or conventional αCTLA4 made no significant difference in any pathways, nor did whether or not NBTXR3 was used. When directly comparing the two quadruple therapies, NBTXR3+XRT+NF-αCTLA4+αPD1 and NBTXR3+XRT+αCTLA4+αPD1 (that is, comparing NF-αCTLA4 and αCTLA4 head-to-head when all other treatment modalities were in play), we observed that the most upregulated genes fell within functional categories predominantly related to innate immune function. These functional categories included the acute phase response, chemotaxis, inflammation, and cell-cell adhesion (**Supplementary Figure 2A**).

We also analyzed the primary tumor for the enrichment of genes indicative of particular immune cell types. We found that macrophage-related genes were significantly enriched in treatment groups containing NF-αCTLA4 relative to both the control group and treatment groups containing αCTLA4, suggesting superior macrophage enrichment by the NF-αCTLA4 antibody compared to conventional αCTLA4 (**Figure 3B**). Several other non-statistically significant trends were also visible. Overall, the XRT+αCTLA4+αPD1, XRT+NF-αCTLA4+αPD1, and NBTXR3+XRT+NF-αCTLA4+αPD1 groups tended to have higher scores in CD8^+^ T cells, dendritic cells (DCs), T_H_1 cells, CD45^+^ cells, Tregs, neutrophils, and macrophages than the control. Moreover, the XRT+αCTLA4+αPD1, XRT+NF-αCTLA4+αPD1, and NBTXR3+XRT+NF-αCTLA4+αPD1 groups tended to have higher scores in DCs, cytotoxic cells, T_H_1 cells, CD45^+^ cells, and macrophages than the NBTXR3+XRT group. In addition, a higher ratio of CD8 T/Treg was observed in NBTXR3+XRT+NF-αCTLA4+αPD1 as compared to NBTXR3+XRT+αCTLA4+αPD1 and XRT+NF-αCTLA4+αPD1 (**Supplementary Figure 3A**). Interestingly, the quadruple therapy with NF-αCTLA4 also led to more abundant total tumor-infiltrating lymphocytes than the control in the primary tumor compared to the quadruple therapy with αCTLA4 (**Supplementary Figure 3A**).

Examining the individual genes that were specifically altered by our treatments (**Figure 3C**, **Supplementary Table 1**), we saw that, compared to NBTXR3+XRT+αCTLA4+αPD1, NBTXR3+XRT+NF-αCTLA4+αPD1 significantly elevated the expression of several chemokine ligands and receptors (*Ccl1*, *Ccl17*, *Cxcl11*, Cxcl14, *Cxcl3*, *Cxcr2*) as well as a smattering of genes primarily involved in innate immunity, including: *Clu*, the gene for clusterin, an extracellular chaperone that promotes clearance of inflammation and injury-induced immune complexes*; >Fut7*, a carbohydrate involved in cell and matrix adhesion that enables leukocyte accumulation at a site of inflammation; *Ifitm1*, an IFN-induced antiviral protein implicated in cell adhesion and control of cell growth and migration; *Lcn2*, a neutrophil-secreted factor that sequesters iron-containing siderophores; and *Spp1*, a cytokine involved in enhancing the production of IFNγ and IL-12 and reducing the production of IL-10 (**Figure 3C**). NF-αCTLA4 also significantly downregulated certain genes involved in the TGFβ pathway, such as *Tgfb2*, *Tgfb3*, *Rora*, and *Rorc* (**Figure 3C**, **Supplementary Figure 3B**). Interestingly, the addition of NBTXR3 to XRT+NF-αCTLA4+αPD1 led to no increase in gene expression (**Figure 3C**, **Supplementary Table 3**) but decreased expression of *Cam*, *Il6*, *Fas*, *Nod2*, *etc.*

**Figure 4:**
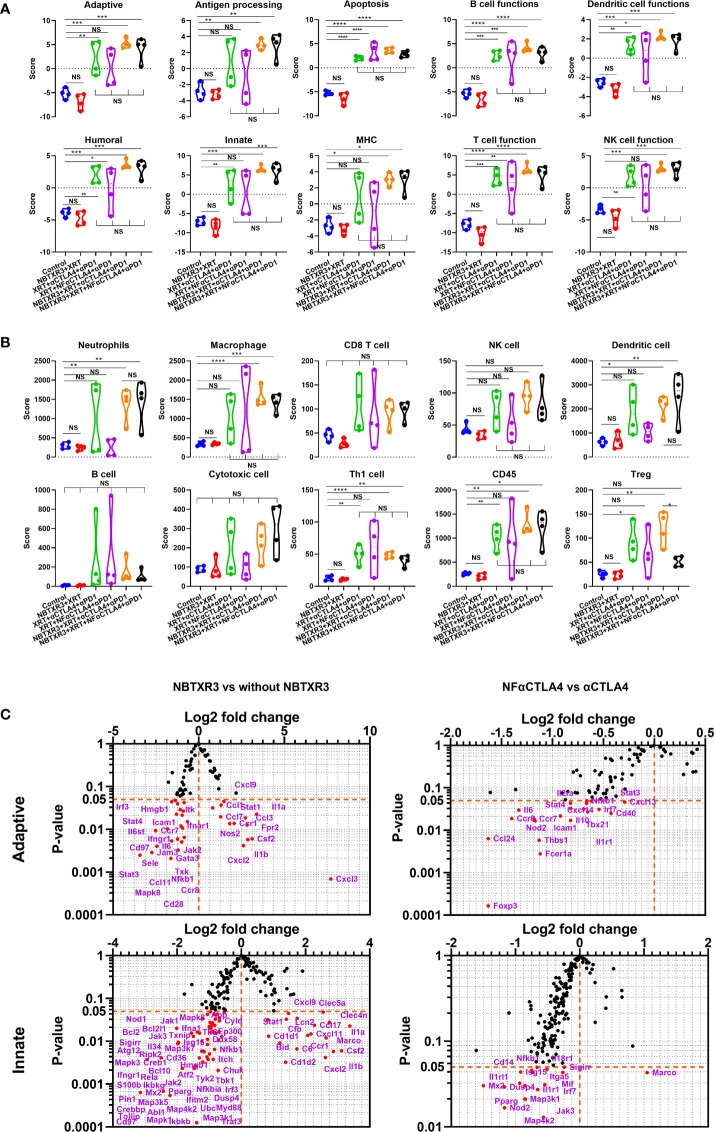
NBTXR3, combined with NF-αCTLA4, reduces Treg abundance and promotes antitumor immune response in the secondary tumor. **(A)** Activities of immune pathways in the secondary tumors. **(B)** Score of immune cell abundance in the secondary tumor. **(C)** Changes in gene expression in adaptive and innate immune pathways. Mice bearing 344SQR tumors were treated with various immunoradiotherapies in **Figure 1A**, and the secondary tumors were harvested on day 18. The RNA extracted from the tumors was analyzed by an nCounter PanCancer Immune Profiling Panel. Data are expressed as means ± SEM. P< 0.05 was considered statistically significant. *P< 0.05, **P< 0.01, ***P< 0.001, ****P< 0.0001, NS, not significant.

As with the primary tumors, in the secondary tumors (**Figure 4A**), all therapies containing CPI resulted in elevated activities in most pathways relative to groups without CPI. Once again, whether the CTLA4 blocker used was NF-αCTLA4 or αCTLA4 here made little difference, as did whether or not NBTXR3 was used. The difference between the primary and secondary tumors was that, unlike in the primary tumors, NBTXR3+XRT did not cause elevated immune activities in the secondary tumor relative to the control. Quadruple therapies had higher scores associated with immune cell populations across the board than the control group (**Figure 4B**). In addition, more abundant tumor-infiltrating lymphocytes were observed in mice treated with combination therapies with CPIs (**Supplementary Figure 3A**). As in the primary tumor, the quadruple therapy with NF-αCTLA4 resulted in a higher CD8/Treg ratio than the quadruple therapy with αCTLA4 in the secondary tumors (**Supplementary Figure 3A**). To our surprise, however, the quadruple therapy group involving NF-αCTLA4 had, on the whole, lower expression of immune-related genes than did the quadruple therapy involving conventional αCTLA4 (**Figure 4C**, **Supplementary Table 2**). Among the downregulated genes were, notably, those related to Treg identity and function. In particular, *Foxp3* expression was significantly downregulated in the NF-αCTLA4 quadruple therapy group compared to the αCTLA4 quadruple therapy group (**Figure 4C**).

Contrary to what was observed in the primary tumors, using the NBTXR3 nanoparticle in concert with CPI significantly upregulated many genes (**Figure 4C**, **Supplementary Figure 3B**, **Supplementary Table 4**). Among the most highly upregulated groups were cytokines (**Supplementary Figure 2C**). These exhibited a bimodal distribution, roughly half upregulated and half downregulated (**Supplementary Figure 2D**). Examining the function of these genes, we found that those cytokine genes that were upregulated with quadruple therapies featuring NBTXR3 – *Il1a, Csf2, Il1b, Spp1, Il12rb1*, and *Il1r2* – were all pro-inflammatory genes. Downregulated cytokine genes also contained pro-inflammatory genes and anti-inflammatory cytokines such as *Il4ra* and *Tgfb3* in the secondary tumors. Also, adding NBTXR3 to XRT+NF-αCTLA4+αPD1 significantly upregulated the *Gzmb* in the secondary tumors.

### Quadruple therapies with NBTXR3 and NF-αCTLA4 generate long-term memory and diverse TCR repertoire against lung cancer

**Figure 5:**
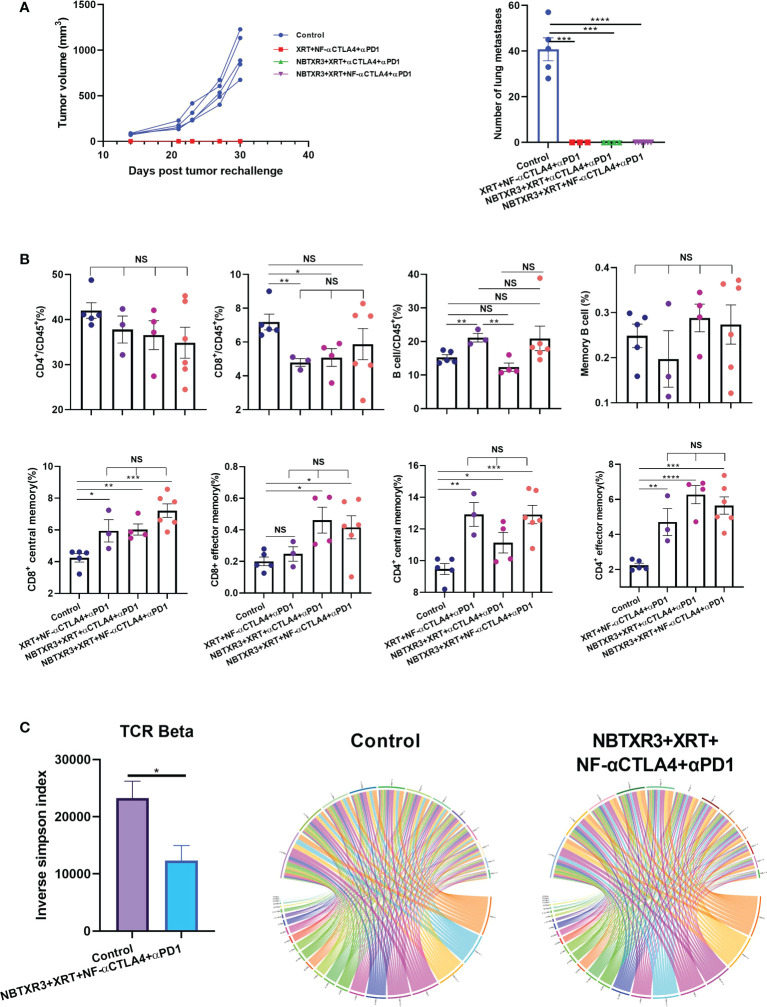
Immunoradiotherapies generate long-term antitumor memory immune response. **(A)** Tumor growth curves and the number of lung metastases of survivors after tumor rechallenge. **(B)** Memory immune cells populations in survivor mice. **(C)** Inverse Simpson index of TCRβ and representative circos plots displaying the pairings of V-J gene families of the TCRβ in survivor mice and control mice. The survivor mice cured by different immunoradiotherapies were rechallenged with 344SQR cells 156 days following their final fraction of radiation. Tumor growth was monitored, and the memory immune cells populations were profiled 21 days post tumor rechallenge. Data are expressed as means ± standard error of the mean (SEM). P< 0.05 was considered statistically significant. *P< 0.05, **P< 0.01, ***P< 0.001, ****P< 0.0001, NS, not significant.

To explore if the mice that survived the initial tumor challenge (**Figure 1C** **)** maintained long-term antitumor immune memory, these survivor mice were rechallenged with 344SQR lung cancer cells 156 days following administration of the last fraction of radiation. As shown in **Figure 5A**, none of the survivor mice from any combination therapies developed tumor after rechallenging, while tumor growth was observed in all the control group mice. In addition, numerous lung metastases were detected in all the control mice, but none was found in the survivor mice (**Figure 5A**).

To understand the immune memory profile of the survivor mice, immune cells, including CD4^+^ T cells, CD8^+^ T cells, and B cells, were analyzed by flow cytometry. As shown in **Figure 5B**, the survivor mice were not significantly different from the control in terms of overall CD4^+^/CD45^+^ ratio, and the control mice had higher percentages of CD8^+^ T cells in their blood than the survivor mice in the XRT+NF-αCTLA4+αPD1 and NBTXR3+XRT+αCTLA4+αPD1 groups. However, all treatment groups had significantly higher percentages of CD4^+^ central memory cells (CD3^+^CD4^+^CD44^+^CD62L^+^), CD8^+^ central memory cells (CD3^+^CD8^+^CD44^+^CD62L^+^), and CD4^+^ effector memory cells (CD3^+^CD4^+^CD44^+^CD62L^-^) than the control (**Figure 5B**, **Supplementary Figure 5**). In addition, both NBTXR3+XRT+NF-αCTLA4+αPD1 and NBTXR3+XRT+αCTLA4+αPD1 had significantly more CD8^+^ effector memory cells (CD3^+^CD8^+^CD44^+^CD62L^-^) than the control (**Figure 5B**, **Supplementary Figure 5**). There was no difference in B cells, either total or memory, between the control mice and the survivors, except for the XRT+NF-αCTLA4+αPD1 group, which exhibited significantly higher total B cell levels. No group of survivor mice boasted any significant elevation in any memory population relative to the other, indicating that each therapy had successfully established a memory population in the surviving mice.

To evaluate the differences in T cell diversity between the treated mice and the control, TCR repertoires from the blood of the control mice and the survivor mice in NBTXR3+XRT+NF-αCTLA4+αPD1 were analyzed. As shown in **Figure 5C**, the survivor mice had a significantly lower inverse Simpson index than the control in the beta chain, indicating that the NBTXR3+NF-αCTLA4-mediated immunoradiotherapy generated a more diverse T cell repertoire.

### Survivor mice in combination therapies of NBTXR3 and NF-αCTLA4 modulate immune gene expression for tumor rejection

**Figure 6:**
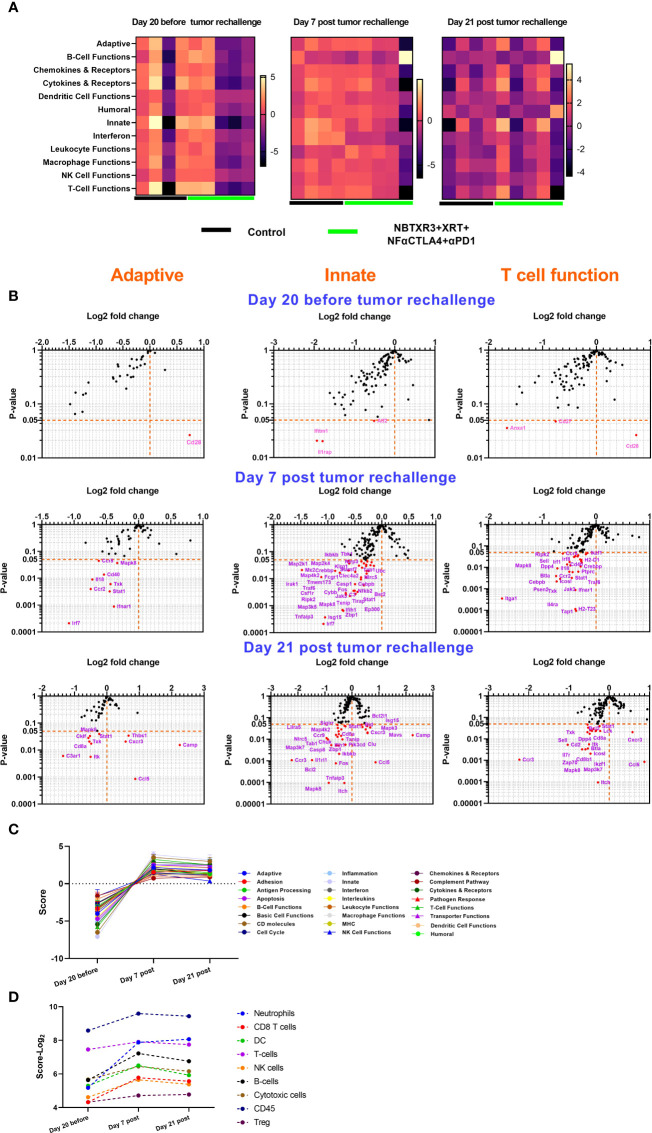
Survivor mice cured by NBTXR3 and NF-αCTLA4 mediated immunoradiotherapy upgrade immune response to prevent tumor relapse. **(A)** Comparison of immune pathway activities of survivor and control mice. **(B)** Differences in immune-related gene expression in survivor mice relative to the control. **(C)** Immune pathway activities in survivor mice before and post tumor rechallenge. **(D)** Immune cell abundance in survivor mice before and post tumor rechallenge. The survivor mice in the NBTXR3+XRT+NF-αCTLA4+αPD1 group were rechallenged with 344SQR cells 156 days post the last radiation fraction. RNA from blood harvested 20 days before, 7 and 21 days post tumor rechallenge were analyzed by NanoString.

To understand how the survivor mice responded to tumor rechallenge at the genetic level, we harvested blood from survivor mice from the NBTXR3+XRT+NF-αCTLA4+αPD1 group 20 days before tumor rechallenge and 7 and 21 days post tumor rechallenge. These blood samples were then analyzed by NanoString, with blood draws from the mice in the control group taken at the same time points for comparison. As shown in **Figure 6A**, 20 days before tumor rechallenge, the mice within the control and NBTXR3+XRT+NF-αCTLA4+αPD1 groups displayed high interindividual variation. Overall, mice in the NBTXR3+XRT+NF-αCTLA4+αPD1 group exhibited lower activities in the various immune pathways measured relative to the control group. However, seven days post tumor rechallenge, survivor mice from the quadruple therapy achieved comparable levels of immune pathway activities to the control. In addition, 21 days post tumor rechallenge, some of the mice in the NBTXR3+XRT+NF-αCTLA4+αPD1 group had higher activities in pathways, such as Adaptive, Chemokines & Receptors, Innate, *etc.* One mouse from the quadruple therapy had much lower level of activities in most of the pathways, but it had much higher activity in B cell function than the control on both day 7 and day 21.

Analysis of individual gene expression in adaptive, innate, and T cell functional pathways revealed that, prior to tumor rechallenge, survivor mice treated with quadruple therapy had significantly altered expression of only five genes: *Cd28*, which was increased, and *Anxa1*, *Cd27*, *Ifitm1*, and *Il1rap*, which were all downregulated (**Figure 6B**). Seven days post tumor rechallenge, mice in the quadruple therapy group exhibited several significantly downregulated genes, including *Irf7*, *Irak*, *Stat1*, *Cd40*, *Isg15*, *Itga1*, *etc.* However, 21 days post-rechallenge, the combination therapy group had downregulated genes such as *C3ar1*, *Cd8a*, *Cklf*, *Ccr3*, *Casp8*, *Sell*, *etc.* upregulated genes, such as *Cxcr3*, *Ccl5*, *Isg15*, *Camp*, *Thbs1*, *Mavs*, *etc.* as compared to the control. By tracking the expression of immune-related genes over time (**Figures 6C, D**), it was found that the survivor mice in the NBTXR3+XRT+NF-αCTLA4+αPD1 group markedly upregulated the activities of immune pathways and increased the abundance of immune cells for coping with the recurring cancer cells.

## Discussion

Thanks to the advances in precision radiotherapy and immune checkpoint blockade, immunoradiotherapy has been increasingly used to treat various types of cancers, particularly metastatic ones (27). A successful immunoradiotherapeutic regimen needs to address two critical problems: one is to induce tumor antigen release and subsequent cytotoxic T cell activation; the other is to minimize the immunosuppressive rebound that typically follows the initial inflammatory insult caused by the radiation. Radiotherapy can effectively kill local tumors, releasing tumor antigen and adjuvant signals, thereby priming the immune system for antitumor activity, essentially converting the tumor into an *in situ* vaccine (28). This process can be further enhanced by NBTXR3, a hafnium oxide nanoparticle (29). NBTXR3 initially developed as a radiation enhancer, has been recently utilized for immune priming (17, 18). NBTXR3-mediated radiotherapy not only leads to enhanced direct tumor destruction (29–31), but also can activate the cGAS-STING pathway in cancer cells (32), improve the immunogenic cell death and modulate the immunopeptidome for promoting antitumor immunity (33).

To address immune suppression, CPI is typically used (34). We previously demonstrated that the combination of NBTXR3+XRT+αCTLA4+αPD1 could potently eradicate both local tumors and metastases, resulting in significantly extend survival (19). However, the application of conventional αCTLA4 is limited due to its side effects in patients (35). Encouragingly, a safer and more efficacious version of αCTLA4, called NF-αCTLA4, has been developed and is now undergoing clinical trials (NCT03110107, NCT04785287) (36). This study shows that NF-αCTLA4 is far more effective at promoting antitumor immunity in combination with XRT and curing mice (50% increase) than αCTLA4. Nevertheless, these treatment results are less effective in the absence of NBTXR3. In fact, the addition of NBTXR3 to αCTLA4 or NF-αCTLA4 markedly improved the efficacy of immunoradiotherapy. The addition of NBTXR3 to αCTLA4+XRT+αPD1 or NF-αCTLA4+XRT+αPD1 significantly improved the control of both primary and secondary tumors. In both cases, NBTXR3 addition allowed to increase by 100% the number of cured mice and significantly increased median survival, compared to the same treatment without NBTXR3 (i.e., improvement of survival rate from 37.5% to 75% when combined with NF-αCTLA4 and αPD1). These results demonstrate the strong immunomodulatory potential of NBTXR3.

As shown in our previous studies, NBTXR3 directly enhances the tumoricidal properties of XRT. This has the added effect of promoting antitumor immunity through the upregulation of antitumor immune genes and facilitating intratumoral cytotoxic T cell infiltration (17). The significant upregulation of *Gzmb*, *Il1a*, *Il1b*, and *Cxcl2*, *Ccl1*, *etc.*, by NBTXR3, suggests this nanoparticle activates cytotoxic effector cells but also may aid their infiltration into tumors by favorably regulating chemokines.

It is worth noting that NBTXR3+XRT alone did not promote immune activities in the secondary tumor, demonstrating that the addition of CPIs is essential for creating effective antitumor immunes. Profiling of immune populations within the tumors *via* FACS revealed that both αCTLA4 and NF-αCTLA4 markedly reduced the number of Tregs in both primary and secondary tumors. The significantly lower Treg score from the NanoString in the NBTXR3+XRT+NF-αCTLA4+αPD1 group relative to the NBTXR3+XRT+αCTLA4+αPD1 group demonstrates that NF-αCTLA4 is more efficacious in depleting Tregs. This is a great significance, as Tregs are one of the primary immune suppression populations (37). The reduction in Treg abundance may increase the activation of CD8^+^ T cells observed in the FACS data. In addition, significant downregulation of potent inhibitory genes such as *Foxp3*, *Ctla4*, *Lag3*, *Tgfb1*, *Il10*, and *Vegfa*, *etc.* by NF-αCTLA4 compared to αCTLA4 implies that this second generation αCTLA4 can reduce immune suppression by inhibiting a wide range of immune suppressor genes (38–40). The significantly higher percentages of Gzm B^+^CD8^+^ T cells in the secondary tumors of mice treated with NBTXR3+XRT+NF-αCTLA4+αPD1 compared to those treated with NBTXR3+XRT+αCTLA4+αPD1 demonstrate that NF-αCTLA4 is more effective than conventional αCTLA4 in improving activation of CD8 T cells.

The efficacy of immunoradiotherapy lies not just in its ability to effectively eradicate existing tumors but also in preventing the relapse of cancers. Our data demonstrate that adding NBTXR3 to NF-αCTLA4-mediated immunoradiotherapy can significantly extend survival and initiate long-term immunological memory that effectively inoculates against tumor reoccurrence. This immunity manifests as elevated levels of memory T cells, including central and effector memory cells of CD4^+^ and CD8^+^ lineages. Also of note is that the survivor mice in the NBTXR3+NF-αCTLA4 quadruple therapy exhibited a significantly more diverse T cell repertoire than the untreated mice. Although further testing would be needed to confirm, we speculate that this results from epitope spreading, with tumor neoepitopes being exposed as a result of the combined therapy, prompting the expansion of T cells of multiple specificities.

## Conclusion

In conclusion, the combination of NBTXR3 nanoparticle-enhanced XRT with NF-αCTLA4+αPD1 CPI improved the control of local tumors and metastases, resulting in a statistically significantly higher mice survival rate. The addition of XRT-activated NBTXR3 to NF-αCTLA4+αPD1 therapy was able to significantly promote the activities of a wide range of immune pathways and downregulate the activity of Treg for improved antitumor immune response. In addition, all mice cured by this combination therapy were immunized to prevent tumor re-growth by maintaining a durable and sustained antitumor memory response and a more diverse TCR repertoire. These data cast an encouraging light on future clinical trials exploring NBTXR3 with multiple CPIs.

## Data availability statement

The datasets presented in this study can be found in online repositories. The names of the repository/repositories and accession number(s) can be found below: https://doi.org/10.6084/m9.figshare.21158677.v1.

## Ethics statement

The animal study was reviewed and approved by IACUC, MD Anderson Cancer Center.

## Author contributions

YH, SP, MC, and JW designed the study. YH, AH, and HB performed the experiments. YH, GB, SP, and QW analyzed the data. YH, GB, and SP wrote the manuscript. All of the authors discussed the results and reviewed the manuscript. All authors read and approved the final manuscript.

## Funding

This work was supported by Cancer Center Support (Core) Grant CA016672 and the NIH 1S10OD024977-01 award to The University of Texas MD Anderson Cancer Center; the Goodwin family research fund; the family of M. Adnan Hamed and the Orr Family Foundation to MD Anderson Cancer Center’s Thoracic Radiation Oncology program; an MD Anderson Knowledge Gap award; Nanobiotix.

## Conflict of interest

JWW reports research support from GlaxoSmithKline, Bristol Meyers Squibb, Merck, Nanobiotix, RefleXion Alkermes, Artidis, Mavu Pharma, Takeda, Varian, and Checkmate Pharmaceuticals. JWW serves on the scientific advisory board for Legion Healthcare Partners, RefleXion Medical, MolecularMatch, Merck, AstraZeneca, Aileron Therapeutics, OncoResponse, Checkmate Pharmaceuticals, Mavu Pharma, Alpine Immune Sciences, Ventana Medical Systems, Nanobiotix, China Medical Tribune, GI Innovation, Genentech and Nanorobotics. JWW is on Speaking Engagements for Ventana Medical Systems, US Oncology, Alkermes, and Boehringer Ingelheim. He is co-founder of Healios, MolecularMatch, OncoResponse and serves as an advisor to Astra Zeneca, OncoResponse, Merck, MolecularMatch, Incyte, Aileron and Nanobiotix. JWW holds stock or ownership in Alpine Immune Sciences, Checkmate Pharmaceuticals, Healios, Mavu Pharma, Legion Healthcare Partners, MolecularMatch, Nanorobotics, OncoResponse, and RefleXion. JWW has accepted honoraria in the form of travel costs from Nanobiotix, RefleXion, Varian, Shandong University, The Korea Society of Radiology, Aileron Therapeutics and Ventana. JWW has the following patents; MP470 amuvatinib, MRX34 regulation of PDL1, XRT technique to overcome immune resistance. MD Anderson Cancer Center has a trademark for XRTTM. SP and JS are employees of Nanobiotix.

The remaining authors declare that the research was conducted in the absence of any commercial or financial relationships that could be construed as a potential conflict of interest.

This study received funding from Nanobiotix. The funder had the following involvement with the study: experiment design, data analysis, and manuscript writing.

## Publisher’s note

All claims expressed in this article are solely those of the authors and do not necessarily represent those of their affiliated organizations, or those of the publisher, the editors and the reviewers. Any product that may be evaluated in this article, or claim that may be made by its manufacturer, is not guaranteed or endorsed by the publisher.

## Supplementary material

Supplementary Figure 1
**(A)** Individual curves of primary and secondary tumors treated with various combination therapies. **(B)** Tumor growth curves and survival rates of mice treated with various combination therapies, in which NF-αCTLA4 was intratumorally injected on day 7 (IT1) or days 7 and 11 (IT2) into the primary tumors.

Supplementary Figure 2 Alteration of immune pathway function by NF-αCTLA4 and NBTXR3. Raw transcript abundance was determined using the nCounter MAX Analysis System, as described in the methods. The average log_2_ fold-change of each gene was determined in , . Genes significantly up- or downregulated were manually assigned to the functional group. **(A)** Functional groups of genes significantly up- or downregulated within primary tumors between NBTXR3+XRT+NF-αCTLA4+αPD1 and NBTXR3+XRT+αCTLA4+αPD1. **(B)** Functional groups of genes significantly up- or downregulated within secondary tumors between treatment groups containing NF-αCTLA4 and those without. **(C)** Functional groups of genes significantly up- or downregulated within secondary tumors between NBTXR3+XRT+NF-αCTLA4+αPD1 and XRT+NF-αCTLA4+αPD1. **(D)** Genes coding for cytokines or their respective receptors that were significantly up- or downregulated within secondary tumors between treatment groups containing NBTXR3 and those without.

Supplementary Figure 3 NBTXR3, in combination with NF-αCTLA4, alters immune-related gene expression in primary tumors. **(A)** Relative immune cells score in the primary tumors. **(B)** Changes in the expression of immune-related genes induced by NF-αCTLA4 or NBTXR3. Mice bearing 344SQR tumors were treated with various immunoradiotherapies in , and the primary tumors were harvested on day 18. The RNA extracted from the tumors was analyzed by a nCounter PanCancer Immune Profiling Panel.

Supplementary Figure 4 NBTXR3, in combination with NF-αCTLA4, alters immune-related gene expression in secondary tumors. **(A)** Relative immune cells score in the secondary tumors. **(B)** Changes in the expression of immune-related genes induced by NF-αCTLA4 or NBTXR3. Mice bearing 344SQR tumors were treated with various immunoradiotherapies in , and the secondary tumors were harvested on day 18. The RNA extracted from the tumors was analyzed by a nCounter PanCancer Immune Profiling Panel.

Supplementary Figure 5 Representative flow cytometry graphs of memory T cells in the blood. Memory T cells populations 21 days post tumor rechallenge were profiled. The survivor mice cured by different immunoradiotherapies were rechallenged with 344SQR cells 156 days following their final fraction of radiation. Naïve mice were also challenged with the same number of 344SQR cells and served as control.
