# Supplementary figures and images for: NBTXR3 improves the efficacy of immunoradiotherapy combining nonfucosylated anti-CTLA4 in an anti-PD1 resistant lung cancer model
Source: Front Immunol. 2022 Nov 3;13:1022011. doi: 10.3389/fimmu.2022.1022011 (PMC9669748; doi:10.3389/fimmu.2022.1022011)

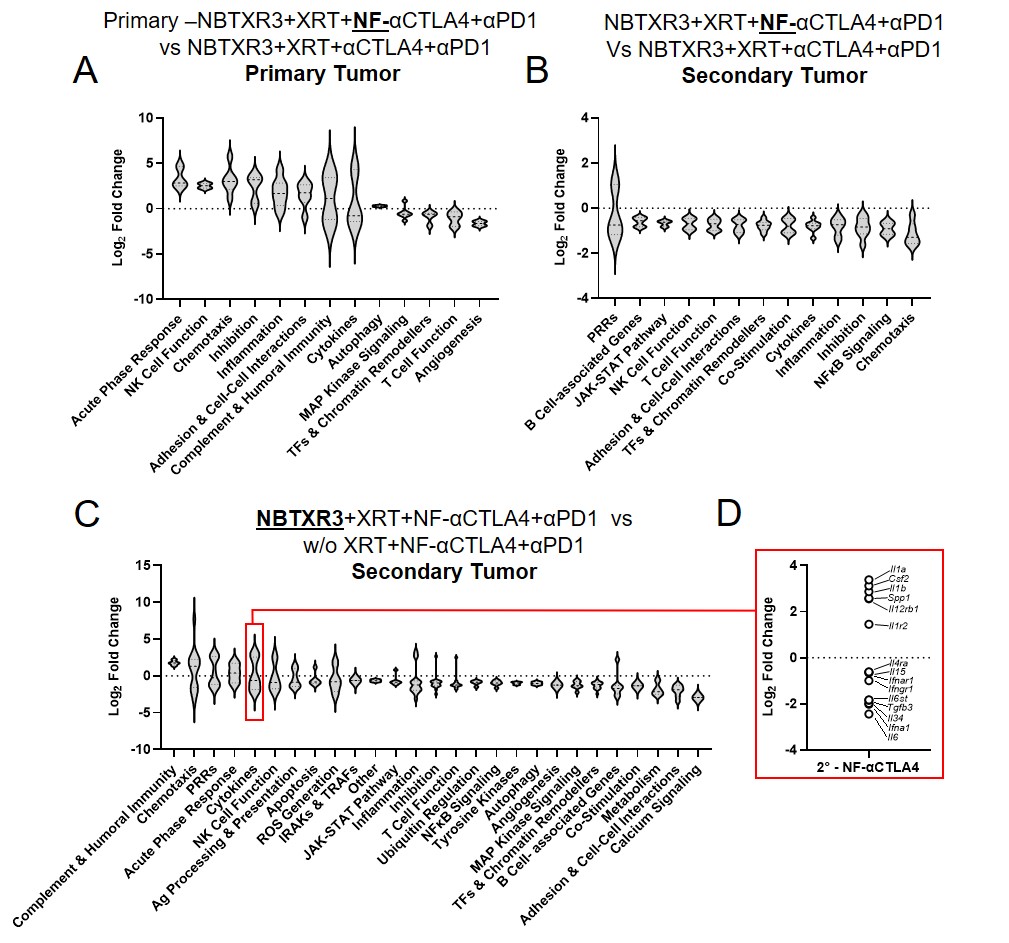

Supplement: Supplementary Figure 2 — Alteration of immune pathway function by NF-αCTLA4 and NBTXR3. Raw transcript abundance was determined using the nCounter MAX Analysis System, as described in the methods. The average log2 fold-change of each gene was determined in , . Genes significantly up- or downregulated were manually assigned to the functional group. (A) Functional groups of genes significantly up- or downregulated within primary tumors between NBTXR3+XRT+NF-αCTLA4+αPD1 and NBTXR3+XRT+αCTLA4+αPD1. (B) Functional groups of genes significantly up- or downregulated within secondary tumors between treatment groups containing NF-αCTLA4 and those without. (C) Functional groups of genes significantly up- or downregulated within secondary tumors between NBTXR3+XRT+NF-αCTLA4+αPD1 and XRT+NF-αCTLA4+αPD1. (D) Genes coding for cytokines or their respective receptors that were significantly up- or downregulated within secondary tumors between treatment groups containing NBTXR3 and those without. [file Image_2.jpeg]

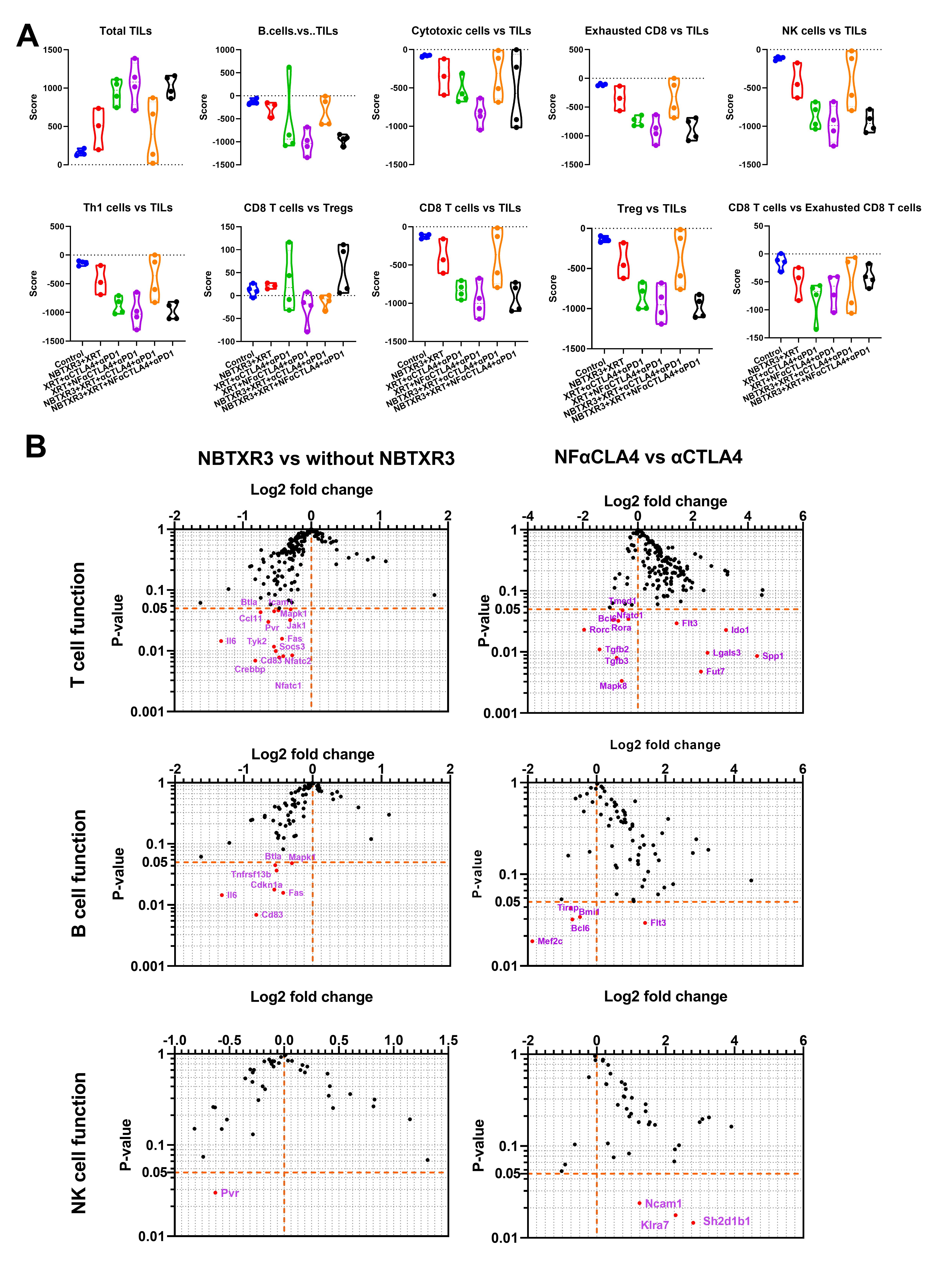

Supplement: Supplementary Figure 3 — NBTXR3, in combination with NF-αCTLA4, alters immune-related gene expression in primary tumors. (A) Relative immune cells score in the primary tumors. (B) Changes in the expression of immune-related genes induced by NF-αCTLA4 or NBTXR3. Mice bearing 344SQR tumors were treated with various immunoradiotherapies in , and the primary tumors were harvested on day 18. The RNA extracted from the tumors was analyzed by a nCounter PanCancer Immune Profiling Panel. [file Image_3.jpeg]

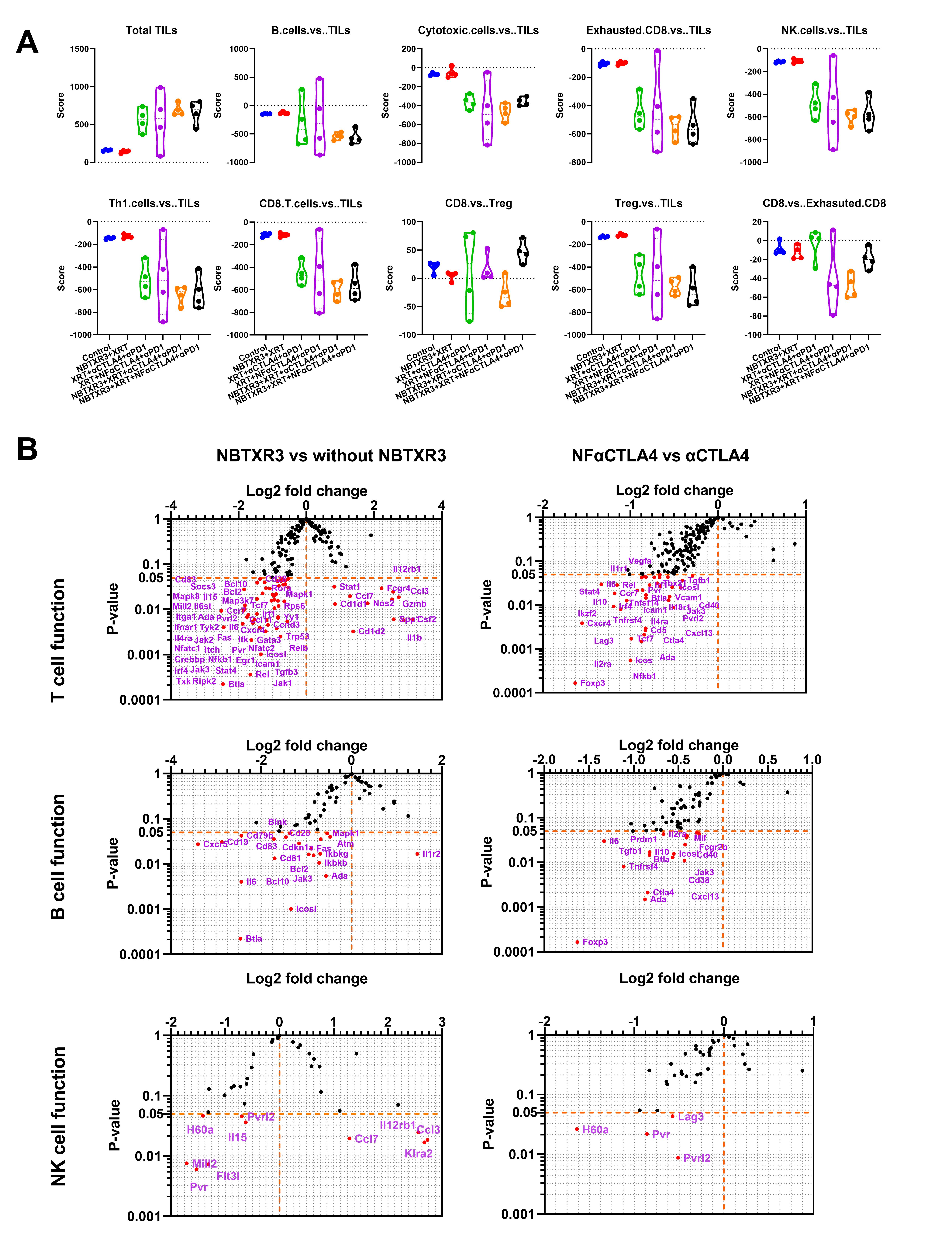

Supplement: Supplementary Figure 4 — NBTXR3, in combination with NF-αCTLA4, alters immune-related gene expression in secondary tumors. (A) Relative immune cells score in the secondary tumors. (B) Changes in the expression of immune-related genes induced by NF-αCTLA4 or NBTXR3. Mice bearing 344SQR tumors were treated with various immunoradiotherapies in , and the secondary tumors were harvested on day 18. The RNA extracted from the tumors was analyzed by a nCounter PanCancer Immune Profiling Panel. [file Image_4.jpeg]

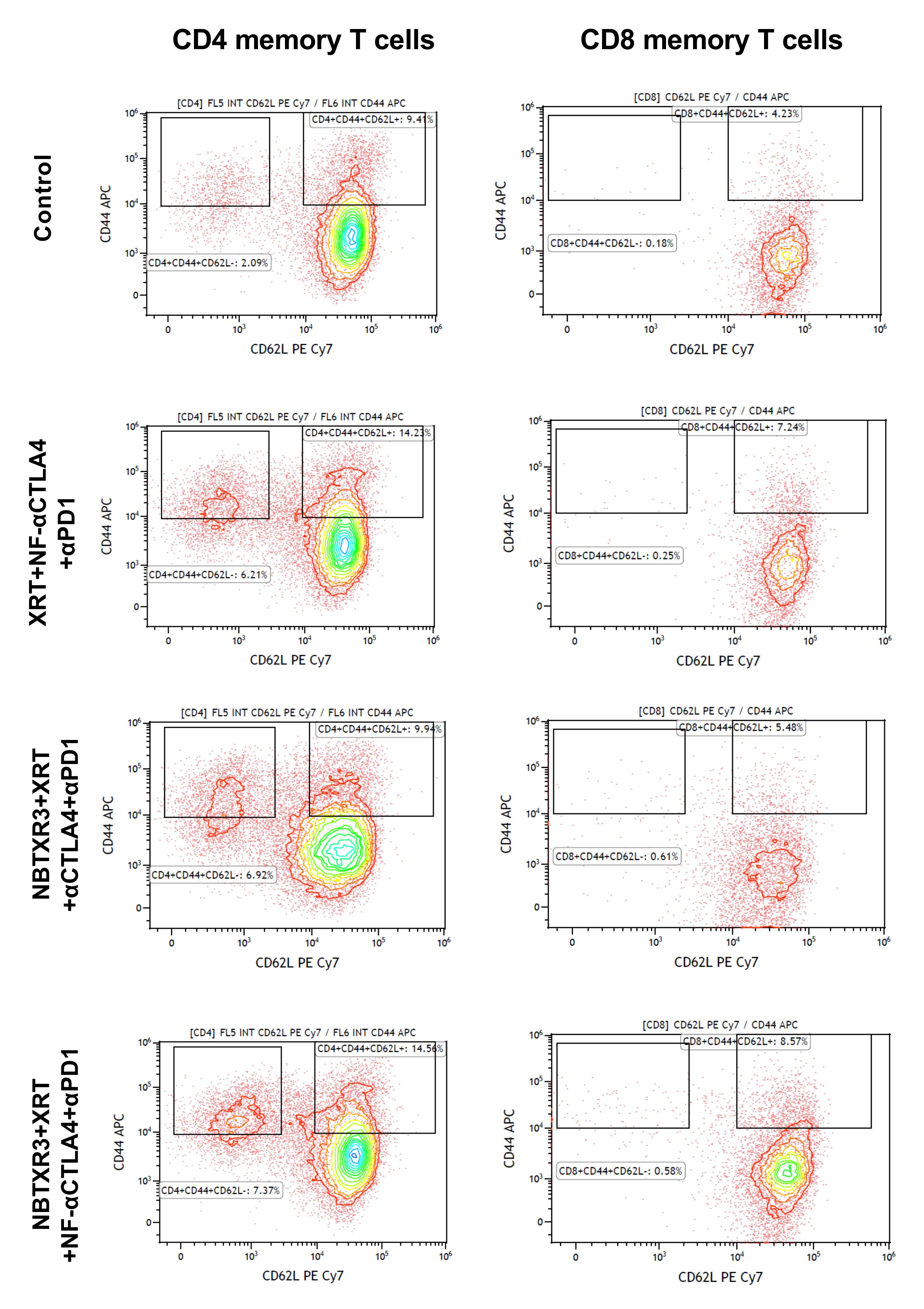

Supplement: Supplementary Figure 5 — Representative flow cytometry graphs of memory T cells in the blood. Memory T cells populations 21 days post tumor rechallenge were profiled. The survivor mice cured by different immunoradiotherapies were rechallenged with 344SQR cells 156 days following their final fraction of radiation. Naïve mice were also challenged with the same number of 344SQR cells and served as control. [file Image_5.jpeg]
