# Supplementary material for: NBTXR3 improves the efficacy of immunoradiotherapy combining nonfucosylated anti-CTLA4 in an anti-PD1 resistant lung cancer model
Source: Front Immunol. 2022 Nov 3;13:1022011. doi: 10.3389/fimmu.2022.1022011 (PMC9669748; doi:10.3389/fimmu.2022.1022011)
Supplement: Supplementary file 6 [file Table_1.docx]

SUPPLEMENTAL TABLE 1: Genes significantly differentially regulated between NBTXR3+XRT+NF-αCTLA4+αPD1 and NBTXR3+XRT+αCTLA4+αPD1 treatment groups in the primary tumor.

| **Gene** | **Log 2 Fold- Change** | **P-Value** | **Full Name** | **Notable Aliases** | **Function** |
| --- | --- | --- | --- | --- | --- |
| ***Acute Phase Response*** | | | | | |
| *Clu* | 2.84 | 0.0133 | Clusterin | Apolipoprotein J; Ku70-binding protein 1 (KUB1) | Extracellular chaperone that promotes clearance of inflammation and injury-induced immune complexes; protects cells against apoptosis and against cytolysis by complement |
| *Dmbt1* | 4.69 | 0.00901 | Deleted in malignant brain tumors 1 | Hensin; muclin; salivary agglutinin; glycoprotein 340 | Function unknown; may play roles in mucosal defense, cellular immune defense, epithelial differentiation, liver regeneration, cell fate and differentiation, taste, and others |
| *Lcn2* | 2.52 | 0.0139 | Lipocalin 2 | Neutrophil gelatinase-associated lipocalin (NGAL) | Neutrophil-secreted factor that sequesters iron-containing siderophores; also functions as a growth factor |
| ***Adhesion & Cell-Cell Interactions*** | | | | | |
| *Fut7* | 2.29 | 0.00476 | Fucosyltransferase 7 | Selectin-ligand synthase | Participates in the biosynthesis of the sialyl Lewis X (sLe(x)), a carbohydrate involved in cell and matrix adhesion that enables leukocyte accumulation at a site of inflammation |
| *Ifitm1* | 2.95 | 0.0193 | Interferon-induced transmembrane protein 1 | CD225 | IFN-induced antiviral protein implicated in cell adhesion and control of cell growth and migration |
| *Itga5* | 1.24 | 0.0354 | Integrin alpha 5 |  | Pairs with ITGB1 to form a receptor for fibronectin and IL-1β |
| *Jam3* | -0.918 | 0.0422 | Junctional adhesion molecule C |  | Immunoglobulin that mediates tight junctions between endothelial cells; mediates transepithelial migration of PMNs; promotes chemotaxis of vascular endothelial cells and stimulates angiogenesis |
| *Lgals3* | 2.53 | 0.00971 | Galectin 3 |  | Galactose-specific lectin that binds IgE; involved in acute inflammatory responses, including neutrophil activation and adhesion, chemoattraction of monocytes macrophages, opsonization of apoptotic neutrophils, and activation of mast cells |
| *Ncam1* | 1.24 | 0.0239 | Neural cell adhesion molecule 1 | CD56 | Cell adhesion molecule involved in the expansion of T-, B-, and NK cells; potentiates signal transduction by interacting with fibroblast growth factor receptors, N-cadherin, and other components of the extracellular matrix; required for effecient cytotoxic cell killing in NK cells |
| ***Angiogenesis*** | | | | | |
| *Mef2c* | -1.87 | 0.0186 | Myocyte enhancer factor 2c |  | Transcriptional activator that binds specifically to the MEF2 element present in the regulatory regions of many muscle-specific genes; controls cardiac morphogenesis and myogenesis, and is also involved in vascular development; required for B cell survival and proliferation in response to BCR stimulation, efficient IgG1 antibody responses to T cell-dependent antigens, and for normal induction of germinal center B cells |
| *Tgfb2* | -1.39 | 0.011 | Transforming growth factor beta 2 | Cetermin; polyergin | Multifunctional protein that regulates various processes such as angiogenesis and heart development |
| ***Autophagy*** | | | | | |
| *Atg12* | 0.221 | 0.0459 | Autophagy related 12 |  | Pairs with ATG5 to promote the extension of the phagophoric membrane in autophagic vesicles |
| *Irgm2* | 0.371 | 0.0374 | Immunity-related GTPase family M member 2 | Interferon-inducible protein 1 (IFI1) | Function not fully known, but most likely regulates autophagy and pro-inflammatory cytokine production |
| ***B Cell-associated Genes*** | | | | | |
| *Mef2c* | -1.87 | 0.0186 | Myocyte enhancer factor 2c |  | Transcriptional activator that binds specifically to the MEF2 element present in the regulatory regions of many muscle-specific genes; controls cardiac morphogenesis and myogenesis, and is also involved in vascular development; required for B cell survival and proliferation in response to BCR stimulation, efficient IgG1 antibody responses to T cell-dependent antigens, and for normal induction of germinal center B cells |
| ***Chemotaxis*** | | | | | |
| *Ccl1* | 3.21 | 0.015 | C-C motif chemokine ligand 1 | T lymphocyte-secreted protein I-309; small inducible cytokine A1 | Chemoattractant for monocytes but not neutrophils; binds to CCR8 |
| *Cxcr2* | 3.5 | 0.0133 | C-X-C motif chemokine receptor 2 | CD182; IL-8 receptor B | Receptor for IL-8 and CXCL3; powerful chemoattractant for neutrophils |
| *Ccl17* | 2.79 | 0.0406 | C-C motif chemokine ligand 17 |  | Chemoattractant ligand for CCR4 and -8; attracts T cells |
| *Cxcl11* | 2.5 | 0.0442 |  |  | Dominant ligand for CXCR3; attracts activated T cells; strongly induced by IFNγ |
| *Cxcl14* | 1.16 | 0.0459 | C-X-C motif chemokine ligand 14 | Bolekine; macrophage inflammatory protein 2-gamma (MIP2γ) | Potent chemoattractant for neutrophils |
| *Cxcl3* | 5.74 | 0.0493 | C-X-C motif chemokine ligand 3 | GRO3 oncogene | Ligand for CXCR2; attracts neutrophils |
| ***Complement & Humoral Immunity*** | | | | | |
| *Cfi* | 3.42 | 0.00863 | Complement factor I | Konglutinogen-activating factor (KAF) | Serine protease that inhibits all three complement pathways by inactivating C3b and C4b |
| *Cd55* | -1.18 | 0.0107 | Cluster of differentiation 55 | Complement decay-accelerating factor | Cell surface glycoprotein that interacts with surface-bound C4b and inhibits its conversion of C2 to C2b |
| ***Cytokines*** | | | | | |
| *Spp1* | 4.33 | 0.00858 | Secreted phosphoprotein 1 | Osteopontin | Cytokine involved in enhancing production of IFNγ and IL-12 and reducing production of IL-10 |
| *Tgfb2* | -1.39 | 0.011 | Transforming growth factor beta 2 | Cetermin; polyergin | Multifunctional protein that regulates various processes such as angiogenesis and heart development |
| *Tgfb3* | -0.767 | 0.00802 | Transforming growth factor beta 3 |  | Multifunctional protein that regulates embryogenesis and cell differentiation |
| ***Inflammation*** | | | | | |
| *Clu* | 2.84 | 0.0133 | Clusterin | Apolipoprotein J; Ku70-binding protein 1 (KUB1) | Extracellular chaperone that promotes clearance of inflammation and injury-induced immune complexes; protects cells against apoptosis and against cytolysis by complement |
| *Irgm2* | 0.371 | 0.0374 | Immunity-related GTPase family M member 2 | Interferon-inducible protein 1 (IFI1) | Function not fully known, but most likely regulates autophagy and pro-inflammatory cytokine production |
| *Isg20* | 1.69 | 0.0482 | Interferon-stimulated gene 20 |  | IFN-induced antiviral exoribonuclease that acts on ssRNA with minor activity towards ssDNA |
| *Itga5* | 1.24 | 0.0354 | Integrin alpha 5 |  | Pairs with ITGB1 to form a receptor for fibronectin and IL-1β |
| *Lgals3* | 2.53 | 0.00971 | Galectin 3 |  | Galactose-specific lectin that binds IgE; involved in acute inflammatory responses, including neutrophil activation and adhesion, chemoattraction of monocytes macrophages, opsonization of apoptotic neutrophils, and activation of mast cells |
| *Mavs* | -0.456 | 0.0206 | Mitochondrial antiviral signaling protein | IFNβ promoter stimulator protein 1 (ISP-1) | Intermediary protein involved in the nonclassical inflammasome pathway; acts downstream of DDX58 and IFIH1, leading to the activation of NFκB, IRF3, and IRF7, and the subsequent induction of IFNβ and RANTES |
| *Spp1* | 4.33 | 0.00858 | Secreted phosphoprotein 1 | Osteopontin | Cytokine involved in enhancing production of IFNγ and IL-12 and reducing production of IL-10 |
| ***Inhibition*** | | | | | |
| *Bcl2l1* | 0.612 | 0.0499 | B cell lymphoma 2 like 1 | Protein phosphatase 1 | Potent inhibitor of caspase-mediated cell death |
| *Cfi* | 3.42 | 0.00863 | Complement factor I | Konglutinogen-activating factor (KAF) | Serine protease that inhibits all three complement pathways by inactivating C3b and C4b |
| *Ido1* | 3.2 | 0.0228 | Indoleamine 2,3-dioxygenase 1 |  | Initiates catabolism of tryptophan; limits immunopathology by inhibiting T cell division |
| ***Macrophage-associated Genes*** | | | | | |
| *Mst1r* | 1.42 | 0.0124 | Macrophage-stimulating 1 receptor | CD136; RON; protein tyrosine kinase 8 (PTK8) | Transduces intracellular signals upon binding to MST1 ligand; regulates many physiological processes including cell survival, migration and differentiation |
| ***MAP Kinase Signaling*** | | | | | |
| *Ecsit* | -1.32 | 0.0171 | Evolutionarily conserved signaling intermediate in Toll pathway |  | Adapter protein of the Toll-like and IL-1 receptor signaling pathways; involved in the activation of NFκB *via* MAP3K1; promotes proteolytic activation of MAP3K1; involved in the BMP signaling pathway. |
| *Mapk3* | 0.878 | 0.0187 | Mitogen-activated protein kinase 3 | Extracellular signal-regulated kinase 1 (ERK1) | Serine/threonine kinase that acts as an essential component of the MAP kinase signal transduction pathway |
| *Mapk8* | -0.586 | 0.00335 | Mitogen-activated protein kinase 8 | c-Jun N-terminal kinase 1 (JNK1); Stress-activated protein kinase 1c (SAPK1) | Serine/threonine-protein kinase involved in various processes such as cell proliferation, differentiation, migration, transformation and programmed cell death; phosphorylates a number of transcription factors, primarily components of AP-1 such as JUN, JDP2, and ATF2, thus regulating AP-1 transcriptional activity; promotes stressed cell apoptosis by phosphorylating key regulatory factors including p53/TP53 and Yes-associates protein YAP1; required for Th1 differentiation |
| *Mapk14* | -0.52 | 0.0405 | Mitogen-activated protein kinase 14 |  | One of the four p38 MAPKs; key kinase in the cascades of cellular responses evoked by extracellular stimuli such as proinflammatory cytokines |
| *Map2k2* | -0.639 | 0.0334 | Mitogen-activated protein kinase kinase 2 | MAPK/ERK kinase 2 (MEK2) | Catalyzes the concomitant phosphorylation of a threonine and a tyrosine residue in a TQY sequence located in MAP kinases; activates ERK1 and -2 |
| *Map2k4* | -0.795 | 0.0199 | Mitogen-activated protein kinase kinase 4 | MAPK/ERK kinase 4 (MEK4); c-Jun N-terminal kinase kinase 1 (JNKK1) | Dual specificity protein kinasethat acts as an essential component of the stress-activated protein/c-Jun N-terminal kinase (SAP/JNK) signaling pathway |
| *Mapkapk2* | -0.328 | 0.00667 | MAP kinase-activated protein kinase 2 |  | Serine/threonine-protein kinase involved in cytokine production, endocytosis, reorganization of the cytoskeleton, cell migration, cell cycle control, chromatin remodeling, DNA damage response, and transcriptional regulation |
| ***NK Cell Function*** | | | | | |
| *Klra7* | 2.8 | 0.0146 | Killer cell lectin-like receptor, subfamily A, member 7 | LGL-1; Ly49G | Potential triggering molecule on murine NK cells |
| *Sh2d1b1* | 2.29 | 0.0177 | SH2 domain-containing protein 1B | EAT2 | Cytoplasmic adapter regulating receptors of the SLAM family; stimulates polarization of the microtubule-organizing center and cytotoxic granules toward the NK cell synapse |
| ***Pattern Recognition Receptors*** | | | | | |
| *Tirap* | -0.749 | 0.0423 | TIR domain-containing adaptor protein |  | Adaptor protein involved in TLR2 and TLR4 signaling; acts *via* IRAK2 and TRAF6, leading to the activation of NFκB, MAPK1, MAPK3 and JNK, and resulting in cytokine secretion and the inflammatory response; positively regulates the production of TNFα and IL-6. |
| ***ROS Generation*** | | | | | |
| *Txnip* | -0.7 | 0.00572 | Thioredoxin interacting protein |  | Thiol-oxidoreductase; protects cells from oxidative stress by inhibiting thioredoxin |
| ***T Cell Function*** | | | | | |
| *Nfatc1* | -0.341 | 0.0346 | Nuclear factor of activated T cells, cytoplasmic 1 |  | Inducible nuclear component of the NFAT TF complex; mediates induction of IL-2 and IL-4 in T cells |
| *Rora* | -0.899 | 0.0334 | Retinoic acid receptor-related orphan receptor A |  | Nuclear receptor that binds hormone response elements upstream of several genes to enhance the expression of those genes |
| *Rorc* | -1.95 | 0.0231 | Retinoic acid receptor-related orphan receptor C |  | Plays a key role, downstream of IL-6 and TGFβ, and synergistically with RORA, for lineage specification of uncommitted CD4+ T helper cells into Th17 cells; may inhibit the expression of Fas ligand and IL-2; may also play a role in the pre-TCR activation cascade leading to the maturation of α/β T cells |
| ***Transcription Factors & Chromatin Remodellers*** | | | | | |
| *Bcl6* | -0.706 | 0.0322 | B cell lymphoma 6 transcriptional repressor |  | Zing finger TF required for GC and memory formation in both B and T cells |
| *Bmi1* | -0.483 | 0.0342 | B lymphoma Mo-murine lymphoma virus insertion region 1 homolog | Polycomb group RING finger protein 4 (PCGF4) | Major component of the polycomb group complex 1 an essential epigenetic repressor of multiple regulatory genes |
| *Hmgb1* | -0.499 | 0.0125 | High-mobility group box 1 |  | Remodels chromatin to make DNA more available for transcription |
| *Mef2c* | -1.87 | 0.0186 | Myocyte enhancer factor 2c |  | Transcriptional activator that binds specifically to the MEF2 element present in the regulatory regions of many muscle-specific genes; controls cardiac morphogenesis and myogenesis, and is also involved in vascular development; required for B cell survival and proliferation in response to BCR stimulation, efficient IgG1 antibody responses to T cell-dependent antigens, and for normal induction of germinal center B cells |
| ***Tyrosine Kinases*** | | | | | |
| *Mst1r* | 1.42 | 0.0124 | Macrophage-stimulating 1 receptor | CD136; RON; protein tyrosine kinase 8 (PTK8) | Transduces intracellular signals upon binding to MST1 ligand; regulates many physiological processes including cell survival, migration and differentiation |
| ***Other*** | | | | | |
| *Flt3* | 1.41 | 0.0294 | Fetal liver kinase 3 | CD135; stem cell tyrosine kinase 1 (STK1) | Cell-surface receptor for the cytokine FLT3LG; regulates differentiation, proliferation, and survival of hematopoietic progenitor cells and DCs |
| *Tmed1* | -0.555 | 0.048 | Transmembrane EMP24 protein transport domain containing 1 | Tp24 | IL1RL1-interacting protein with potential roles in vesicular protein trafficking in the early secretory pathway, incorporation of secretory cargo molecules into transport vesicles, and cytosolic vesicle coat formation |

**Supplementary Table 2: Genes significantly differentially regulated between NBTXR3+XRT+NF-αCTLA4+αPD1 and NBTXR3+XRT+αCTLA4+αPD1 treatment groups in the secondary tumor.**

| **Gene** | **Log 2 Fold-Change** | **P-Value** | **Full Name** | **Notable Aliases** | **Function** |
| --- | --- | --- | --- | --- | --- |
| ***Adhesion & Cell-Cell Interactions*** | | | | | |
| *Icam1* | -0.922 | 0.0216 | Intracellular adhesion molecule 1 |  | Cell surface glycoprotein that serves as strong adhesive ligand for LFA-1; important for leukocyte mobility and costimulation |
| *Itga5* | -0.509 | 0.047 | Integrin alpha 5 |  | Pairs with ITGB1 to form a receptor for fibronectin and IL-1β |
| *Thbs1* | -1.13 | 0.0058 | Thrombospondin 1 |  | Adhesive glycoprotein that mediates cell-to-cell and cell-to-matrix interactions; ligand for CD36 |
| *Vcam1* | -0.523 | 0.0256 | Vascular cell adhesion molecule 1 | CD106 | Endothelial-cell adhesion molecule that binds to ITGA4/ITGB1 on leukocytes and mediates both adhesion and signal transduction |
| ***Angiogenesis*** | | | | | |
| *Vegfa* | -0.869 | 0.0425 | Vascular endothelial growth factor A |  | Glysosylated mitogen that promotes vascular permeability, vasculogenesis, angiogenesis, and cell migration |
| ***Antigen Processing & Presentation*** | | | | | |
| *H60a* | -1.64 | 0.0264 | Histocompatibility 60a |  | Enables NK cell lectin-like receptor binding activity |
| ***B Cell-associated Genes*** | | | | | |
| *Btla* | -0.822 | 0.0146 | B and T lymphocyte attenuator | CD272 | Inhibitory cell surface protein that inhibits T cell function by binding to B7H4 and TNFRSF14 |
| *Cd5* | -0.828 | 0.00265 | Cluster of differentiation 5 | LEU1 | Type-I transmembrane glycoprotein found on the surface of T and B cells; may act as a receptor in regulating T cell proliferation |
| *Fcgr2b* | -0.399 | 0.0391 | Fc fragment of immunoglobulin gamma receptor IIb | CD32 | Low affinity receptor for the Fc region of complexed or aggregated γ-Igs; involved in a variety of effector and regulatory functions such as phagocytosis of immune complexes and modulation of antibody production by B cells |
| *Icosl* | -0.551 | 0.0155 | Inducible T cell costimulator ligand | CD275 | Ligand for T cell-specific co-receptor ICOS; also induces B cell proliferation and plasma cell differentiation |
| *Prdm1* | -0.419 | 0.0403 | Positive regulatory domain I-binding factor | B lymphocyte-induced maturation protein (BLIMP1) | Transcription factor that plays a role in the development, retention, and long-term establishment of T cell, NK cell, and NK-T cells in non-lymphoid organs; drives the maturation of B cell into Ig secreting cells |
| ***Chemotaxis*** | | | | | |
| *Ccl24* | -1.63 | 0.00634 | C-C motif chemokine ligand 24 | Eotaxin-2 | Chemoattractant for resting T cells and eosinophils |
| *Ccr7* | -1.18 | 0.0185 | C-C chemokine receptor type 7 | CD197 | Chemokine receptor that activates B and T cells and promotes their homing to secondary lymphoid organs; also stimulates DC expression of MHC class I and II |
| *Ccr8* | -1.4 | 0.0188 | C-C motif chemokine receptor 8 |  | Receptor for CCL1; may regulate monocyte chemotaxis and thymic cell line apoptosis |
| *Cxcl13* | -0.291 | 0.0464 | C-X-C motif chemokine ligand 13 | BLC, BCA-1 | B cell chemokine induced by type I interferons; participates in germinal center formation |
| *Cxcl14* | -0.82 | 0.0326 | C-X-C motif chemokine ligand 14 | Bolekine; macrophage inflammatory protein 2-gamma (MIP2γ) | Potent chemoattractant for neutrophils |
| *Cxcr4* | -1.55 | 0.00386 | Chemokine receptor CXCR4 | Fusin; CD184 | Alpha-chemokine receptor specific for SDF1 *aka* CXCL12 |
| ***Complement & Humoral Immunity*** | | | | | |
| *Fcer1a* | -1.12 | 0.00278 | Fc epsilon receptor 1a |  | High affinity receptor for IgE; responsible for initiating the allergic response |
| ***Co-Stimulation*** | | | | | |
| *Cd40* | -0.424 | 0.025 | Cluster of differentiation 40 |  | APC-expressed co-stimulatory protein that binds to CD40L on CD4+ T cells, causing activation of both |
| *Icosl* | -0.551 | 0.0155 | Inducible T cell costimulator ligand | CD275 | Ligand for T cell-specific co-receptor ICOS; also induces B cell proliferation and plasma cell differentiation |
| *Tnfrsf4* | -1.11 | 0.00805 | TNF receptor superfamily member 4 | OX40; CD134 | Receptor for TNFSF4/OX40L/GP34; costimulatory molecule implicated in long-term T cell immunity; activates NFκB through its interaction with adaptor proteins TRAF2 and TRAF5; suppresses apoptosis through upregulation of BCL2 |
| *Tnfsf14* | -1.04 | 0.0126 | TNF receptor superfamily member 14 | CD270 | Receptor for four distinct ligands: LIGHT, lymphotoxin-α, BTLA, and CD160, altogether defining a complex stimulatory and inhibitory signaling network; signals *via* the TRAF2-TRAF3 E3 ligase pathway to promote immune cell survival and differentiation; participates in bidirectional cell-cell contact signaling between APCs and lymphocytes; delivers costimulatory signals to T cells, promoting cell proliferation and effector functions; interacts with CD160 on NK cells, enhancing IFNγ production and anti-tumor immune response; upon binding to CD160 on activated CD4+ T cells, downregulates CD28 costimulatory signaling; participates in *cis* or *trans* reactions with BTLA - *cis* interactions seem to promote quiescence; *trans* interactions seem to promote survival |
| ***Cytokines*** | | | | | |
| *Tgfb1* | -0.408 | 0.0361 | Transforming growth factor beta 1 |  | Multifunctional protein that regulates the growth and differentiation of various cell types and is involved in various processes, such as normal development, immune function, microglia function and responses to neurodegeneration; can induce EMT and cell migration in various cell types; frequently acts as an immunosuppressive cytokine in the TME |
| *Il1r1* | -0.655 | 0.0273 | Interleukin 1 receptor type I | CD121a | Receptor for IL-1α and IL-1β; drives several cytokine-induced and inflammatory responses through activation of NFκB and MAPK; recruits TOLLIP, MyD88, IRAK1, and IRAK2 |
| *Il2ra* | -0.665 | 0.0431 | Interleukin 2 receptor subunit alpha | CD25 | Alpha chain of the IL-2 receptor |
| *Il18r1* | -0.728 | 0.0431 | Interleukin 18 receptor 1 | CD218a | Receptor for IL-18 |
| *Il10* | -0.821 | 0.017 | Interleukin 10 | Cytokine synthesis inhibitory factor (CSIF) | Major immunoregulatory cytokine that inhibits production of pro-inflammatory cytokines, including GM-CSF, G-CSF, IL-1α, IL-1β, IL-6, IL-8, and TNFα; also interferes with antigen presentation by reducing expression of MHC class II and co-stimulatory molecules, thereby inhibiting their ability to induce T cell activation |
| *Il4ra* | -0.825 | 0.00293 | Interleukin 4 receptor subunit alpha | CD124 | Alpha chain for the IL-4 and IL-13 receptors; involved in Th2 differentiation and IgE production |
| *Il1rl1* | -0.935 | 0.0323 | Interleukin 1 receptor-like 1 |  | Receptor for IL-33; recruits MyD88, IRAK1, IRAK4, and TRAF6; activates ERK1, ERK2, and MAPK14 |
| *Il6* | -1.33 | 0.0298 | Interleukin 6 |  | Pro-inflammatory cytokine that signals through the JAK and STAT pathways |
| ***Inflammation*** | | | | | |
| *Cd38* | -0.431 | 0.0109 | Cluster of differentiation 38 | ADP-ribosyl cyclase 1 | Synthesizes the second messengers cyclic ADP-ribose and NADPH; appears to play a critical role in inflammation, although its exact immunological function(s) remain(s) poorly defined |
| *Il1r1* | -0.655 | 0.0273 | Interleukin 1 receptor type I | CD121a | Receptor for IL-1α and IL-1β; drives several cytokine-induced and inflammatory responses through activation of NFκB and MAPK; recruits TOLLIP, MyD88, IRAK1, and IRAK2 |
| *Il6* | -1.33 | 0.0298 | Interleukin 6 |  | Pro-inflammatory cytokine that signals through the JAK and STAT pathways |
| *Il18r1* | -0.728 | 0.0431 | Interleukin 18 receptor 1 | CD218a | Receptor for IL-18 |
| *Irf1* | -0.78 | 0.0288 | Interferon regulatory factor 1 |  | Transcriptional regulator that promotes inflammatory innate and adaptive immune responses |
| *Irf4* | -1.19 | 0.00931 | Interferon regulatory factor 4 |  | Transcriptional activator that complexes with BATF and binds ISREs within the promoters of multiple genes involved in inflammation |
| *Irf7* | -0.543 | 0.0311 | Interferon regulatory factor 7 |  | Key transcriptional regulator of type I IFN-dependent immune responses; promotes transcription of IFNα and -β |
| *Isg15* | -0.918 | 0.0437 | Interferon-stimulated gene 15 |  | Ubiquitin-like protein that binds intracellular target proteins upon activation by IFNα or β; can also be secreted to induce NK cell proliferation, act as a chemoattractant for neutrophils, and induce IFNγ upon binding to ITGAL/ITGB2 |
| *Itga5* | -0.509 | 0.047 | Integrin alpha 5 |  | Pairs with ITGB1 to form a receptor for fibronectin and IL-1β |
| *Mif* | -0.264 | 0.0432 | Macrophage migration inhibitory factor | L-dopachrome tautomerase | Pro-inflammatory cytokine that promotes macrophage function through suppression of anti-inflammatory effects of glucocorticoids |
| *Mx2* | -1.5 | 0.0302 | Myxovirus resistance protein 2 |  | IFN-induced dynamin-like GTPase with potent antiviral activity against HIV-1 |
| ***Inhibition*** | | | | | |
| *Btla* | -0.822 | 0.0146 | B and T lymphocyte attenuator | CD272 | Inhibitory cell surface protein that inhibits T cell function by binding to B7H4 and TNFRSF14 |
| *Ctla4* | -0.842 | 0.00212 | Cytotoxic T lymphocyte antigen 4 |  | Inhibitory receptor that blocks CD28 co-stimulation by competitively binding its ligands CD80 and CD86 |
| *Dusp4* | -1.17 | 0.0292 | Dual specificity phosphatase 4 |  | Inactivates ERK1, ERK2, and JNK |
| *Foxp3* | -1.63 | 0.000165 | Forkhead box P3 | DIETER | Master transcription factor for regulatory T cells (Tregs); represses expression of *Il2 and Ifng*; activates expression of *Tnfrsf18*, *Il2ra*, and *Ctla4* |
| *Il10* | -0.821 | 0.017 | Interleukin 10 | Cytokine synthesis inhibitory factor (CSIF) | Major immunoregulatory cytokine that inhibits production of pro-inflammatory cytokines, including GM-CSF, G-CSF, IL-1α, IL-1β, IL-6, IL-8, and TNFα; also interferes with antigen presentation by reducing expression of MHC class II and co-stimulatory molecules, thereby inhibiting their ability to induce T cell activation |
| *Lag3* | -0.572 | 0.0436 | Lymphocyte activating gene 3 | CD223 | Inhibitory receptor on activated T cells; binds to ligands, such as FGL1; constitutively expressed on a subset of regulatory Tregs and contributes to their suppressive function; acts as a negative regulator of plasmacytoid dendritic cell (pDCs) activation |
| *Sigirr* | -0.239 | 0.0499 | Single Ig domain-containing IL-1R-related protein |  | Negative regulator of the Toll-like and IL-1R receptor signaling pathways; attenuates the recruitment of receptor-proximal signaling components to the TLR4 receptor; interferes with the heterodimerization of Il1R1 and IL1RAP |
| *Tgfb1* | -0.408 | 0.0361 | Transforming growth factor beta 1 |  | Multifunctional protein that regulates the growth and differentiation of various cell types and is involved in various processes, such as normal development, immune function, microglia function and responses to neurodegeneration; can induce EMT and cell migration in various cell types; frequently acts as an immunosuppressive cytokine in the TME |
| *Tnfsf14* | -1.04 | 0.0126 | TNF receptor superfamily member 14 | CD270 | Receptor for four distinct ligands: LIGHT, lymphotoxin-α, BTLA, and CD160, altogether defining a complex stimulatory and inhibitory signaling network; signals *via* the TRAF2-TRAF3 E3 ligase pathway to promote immune cell survival and differentiation; participates in bidirectional cell-cell contact signaling between APCs and lymphocytes; delivers costimulatory signals to T cells, promoting cell proliferation and effector functions; interacts with CD160 on NK cells, enhancing IFNγ production and anti-tumor immune response; upon binding to CD160 on activated CD4+ T cells, downregulates CD28 costimulatory signaling; participates in *cis* or *trans* reactions with BTLA - *cis* interactions seem to promote quiescence; *trans* interactions seem to promote survival |
| ***JAK-STAT Pathway*** | | | | | |
| *Jak3* | -0.563 | 0.0129 | Janus kinase 3 |  | Non-receptor tyrosine kinase involved in various processes such as cell growth, development, or differentiation; mediates essential signaling events in both innate and adaptive immunity |
| *Stat3* | -0.616 | 0.0484 | Signal transducer and activator of transcription 3 |  | Transcriptional activator of genes involved in cell growth and apoptosis; activated by JAKs |
| *Stat4* | -0.823 | 0.0435 | Signal transducer and activator of transcription 4 |  | Essential TF for Th1 CD4+ T cell development and IFNγ production; also promotes expression of MyD88 |
| ***MAP Kinase Signaling*** | | | | | |
| *Map3k1* | -0.852 | 0.0212 | Mitogen-activated protein kinase kinase kinase 1 |  | Serine/threonine kinase that activates the ERK and JNK kinase pathways by phosphorylation of MAP2K1 and MAP2K4; also activates CHUK and IKBKB, the central protein kinases of the NFκB pathway |
| *Map4k2* | -0.846 | 0.021 | Mitogen-activated protein kinase kinase kinase kinase 2 |  | Essential component of the MAP kinase signal transduction pathway downstream of TRAF6; upstream activator of the SAP/JNK signaling pathway |
| ***Metabolism*** | | | | | |
| *Ada* | -0.872 | 0.00148 | Adenosine deaminase |  | Key enzyme in purine metabolism; primarily involved in the development and maintenance of the immune system in humans |
| *Pparg* | -0.85 | 0.0303 | Peroxisome proliferator activated receptor gamma |  | Nuclear receptor that binds peroxisome proliferators such as hypolipidemic drugs and fatty acids; once activated binds to specific PPAR response elements (PPRE) and modulates the transcription of its target genes, such as acyl-CoA oxidase, thereby controlling the peroxisomal beta-oxidation pathway of fatty acids |
| ***NFκB Signaling*** | | | | | |
| *Nfkb1* | -0.663 | 0.0497 | Nuclear factor kappa B subunit 1 | p105/p50 | One of the NFκB family TFs; inhibits inflammation |
| *Rel* | -1.15 | 0.0287 | Avian reticuloendotheliosis viral oncogene homolog | c-Rel | One of the NFκB family TFs; important for B cell and Treg development |
| ***NK Cell Function*** | | | | | |
| *Prdm1* | -0.419 | 0.0403 | Positive regulatory domain I-binding factor | B lymphocyte-induced maturation protein (BLIMP1) | Transcription factor that plays a role in the development, retention, and long-term establishment of T cell, NK cell, and NK-T cells in non-lymphoid organs; drives the maturation of B cell into Ig secreting cells |
| *Pvr* | -0.86 | 0.0219 | Poliovirus receptor | CD155 | Mediates NK cell adhesion and triggers NK cell effector functions; binds CD96 and CD226, leading to the formation of a mature immunological synapse between NK cell and target cell |
| *Pvrl2* | -0.51 | 0.00878 | Poliovirus receptor-related protein 2 | Nectin-2 | Variable costimulator/coinhibitor of T cell function, depending on which receptor it binds to: stimulates T cell proliferation and cytokine production upon binding to CD226; inhibits T cell proliferation upon interaction with PVRIG |
| *Tcf7* | -0.989 | 0.0017 | Transcription factor 7 |  | HMG box TF predominantly expressed by T cells that drives their development, although also involved in NK cell development; activates transcription through a Wnt/β-catenin signaling pathway |
| ***Pattern Recognition Receptors*** | | | | | |
| *Cd14* | -0.745 | 0.0458 | Cluster of differentiation 14 |  | PRR that recognizes LPS; mostly found on macrophages |
| *Marco* | 1.05 | 0.0431 | Macrophage receptor with collagenous structure |  | A PRR that recognizes LDL |
| *Nod2* | -1.17 | 0.0167 | Nucleotide-binding oligomerization domain containing 2 |  | PRR specific for muramyl dipeptide (MDP); upon binding to its ligand, recruits RIPK2 and triggers MAPK and NFκB signaling |
| ***T Cell Function*** | | | | | |
| *Icos* | -0.999 | 0.000546 | Inducible T cell costimulator | CD278 | Enhances all basic T cell responses to foreign antigen; essential both for efficient interaction between T and B cells and for normal antibody responses to T cell-dependent Ags |
| *Icosl* | -0.551 | 0.0155 | Inducible T cell costimulator ligand | CD275 | Ligand for T cell-specific co-receptor ICOS; also induces B cell proliferation and plasma cell differentiation |
| *Prdm1* | -0.419 | 0.0403 | Positive regulatory domain I-binding factor | B lymphocyte-induced maturation protein (BLIMP1) | Transcription factor that plays a role in the development, retention, and long-term establishment of T cell, NK cell, and NK-T cells in non-lymphoid organs; drives the maturation of B cell into Ig secreting cells |
| *Tbx21* | -0.692 | 0.0316 | T-box transcription factor 21 |  | Initiates Th1 lineage development from naïve Th precursor cells both by activating Th1 genetic programs and by repressing the opposing Th2 and Th17 genetic programs |
| *Tcf7* | -0.989 | 0.0017 | Transcription factor 7 |  | HMG box TF predominantly expressed by T cells that drives their development, although also involved in NK cell development; activates transcription through a Wnt/β-catenin signaling pathway |
| ***Transcription Factors & Chromatin Remodellers*** | | | | | |
| *Ikzf2* | -0.851 | 0.0459 | IKAROS family zinc finger protein 2 |  | Heamtopoietic cell-specific TF involved in early hematopoietic development |
| *Nfkb1* | -0.663 | 0.0497 | Nuclear factor kappa B subunit 1 | p105/p50 | One of the NFκB family TFs; inhibits inflammation |
| *Prdm1* | -0.419 | 0.0403 | Positive regulatory domain I-binding factor | B lymphocyte-induced maturation protein (BLIMP1) | Transcription factor that plays a role in the development, retention, and long-term establishment of T cell, NK cell, and NK-T cells in non-lymphoid organs; drives the maturation of B cell into Ig secreting cells |
| *Rel* | -1.15 | 0.0287 | Avian reticuloendotheliosis viral oncogene homolog | c-Rel | One of the NFκB family TFs; important for B cell and Treg development |
| *Stat3* | -0.616 | 0.0484 | Signal transducer and activator of transcription 3 |  | Transcriptional activator of genes involved in cell growth and apoptosis; activated by JAKs |
| *Stat4* | -0.823 | 0.0435 | Signal transducer and activator of transcription 4 |  | Essential TF for Th1 CD4+ T cell development and IFNγ production; also promotes expression of MyD88 |
| *Tbx21* | -0.692 | 0.0316 | T-box transcription factor 21 |  | Initiates Th1 lineage development from naïve Th precursor cells both by activating Th1 genetic programs and by repressing the opposing Th2 and Th17 genetic programs |
| *Tcf7* | -0.989 | 0.0017 | Transcription factor 7 |  | HMG box TF predominantly expressed by T cells that drives their development, although also involved in NK cell development; activates transcription through a Wnt/β-catenin signaling pathway |

**Supplementary Table 3: Genes significantly differentially regulated between treatment groups possessing and lacking NBTXR3 in the primary tumor.**

| **Gene** | **Log 2 Fold-Change** | **P-Value** | **Full Name** | **Notable Aliases** |  | **Function** |
| --- | --- | --- | --- | --- | --- | --- |
| *Ifngr1* | -0.359 | 0.0489 | Interferon gamma receptor 1 | CD54 |  | One of the two components of the IFNγ receptor; stimulates activation of the JAK/STAT signaling pathway |
| *Il6* | -1.32 | 0.0145 | Interleukin 6 |  |  | Pro-inflammatory cytokine that signals through the JAK and STAT pathways; induces VEGF to promote angiogenesis |
| *Icam1* | -0.481 | 0.0464 | Intracellular adhesion molecule 1 |  |  | Cell surface glycoprotein that serves as strong adhesive ligand for LFA-1; important for leukocyte mobility and costimulation |
| *Ccl11* | -0.742 | 0.0433 | C-C motif chemokine ligand 11 | Eotaxin |  | Chemoattractant for eosinophils |
| *Cxcl1* | -0.744 | 0.0292 | C-X-C motif chemokine ligand 1 | GRO1 oncogene |  | Chemoattractant ligand for CXCR2; plays a role in inflammation and as a chemoattractant for neutrophils |
| *Nod2* | -0.779 | 0.0411 | Nucleotide-binding oligomerization domain containing 2 |  |  | PRR specific for muramyl dipeptide (MDP); upon binding to its ligand, recruits RIPK2 and triggers MAPK and NFκB signaling |
| *Creb5* | -0.849 | 0.0404 | CAMP responsive element binding protein 5 |  |  | Phosphorylation-dependent transcription factor that stimulates transcription upon binding to the DNA cAMP response element (CRE), which is found in the promoter regions of several immune-related genes, including *Il2*, *Il6*, *Il10*, and *Tnfa*; regulates diverse cellular responses, including proliferation, survival, and differentiation |
| *Camp* | -2.48 | 0.0266 | Cathelicidin antimicrobial peptide |  |  | Polypeptide stored in the lysosomes of macrophages and PMNs that digests phagocytosed cells |

**Supplementary Table 4: Genes significantly differentially regulated between treatment groups possessing and lacking NBTXR3 in the secondary tumor.**

| **Gene** | **Log 2 Fold-Change** | **P-Value** | **Full Name** | **Notable Aliases** | **Function** |
| --- | --- | --- | --- | --- | --- |
| ***Acute Phase Response*** | | | | | |
| *App* | -0.956 | 0.0489 | Amyloid-beta precursor protein |  | A secreted antimicrobial peptide |
| *Lcn2* | 1.74 | 0.0342 | Lipocalin 2 | Neutrophil gelatinase-associated lipocalin (NGAL) | Neutrophil-secreted factor that sequesters iron-containing siderophores; also functions as a growth factor |
| ***Adhesion & Cell-Cell Interactions*** | | | | | |
| *Abl1* | -1.23 | 0.00999 | Abelson tyrosine-protein kinase 1 | Proto-oncogene C-ABL | Plays a role in many key processes linked to cell growth and survival such as cytoskeleton remodeling, cell motility and adhesion, receptor endocytosis, autophagy, DNA damage response, and apoptosis; regulates T cell differentiation by phosphorylating TBX21, leading to its enhancement |
| *Cd97* | -1.23 | 0.00591 | Cluster of differentiation 97 | BL-Ac[F2] | GPCR that promotes granulocyte adhesion and migration; activates T cells *via* binding to CD55; stimulates angiogenesis through binding integrin counterreceptors on endothelial cells |
| *Itga1* | -1.88 | 0.00479 | Integrin subunity alpha 1 | CD49a; Very late activation protein 1 (VLA-1) | Alpha 1 subunit for common integrin receptors; pairs with the β1 subunit to form a cell-surface receptor for collagen and laminin; involved in cell-cell adhesion and may play a role in inflammation and fibrosis |
| *Jam3* | -2.73 | 0.00287 | Junctional adhesion molecule C |  | Immunoglobulin that mediates tight junctions between endothelial cells; mediates transepithelial migration of PMNs |
| *Mill2* | -1.71 | 0.00755 | MHC class I-like protein |  | Heterodimer with β2-microglobulin with MHC class I; orthologous to human MICA and MICB |
| *Sele* | -3.43 | 0.00248 | E-selectin | CD62E; endothelial leukocyte adhesion molecule 1 (ELAM-1) | Cell-surface glycoprotein that mediates in the adhesion of blood neutrophils in cytokine-activated endothelium through interaction with SELPLG/PSGL1 |
| ***Apoptosis*** | | | | | |
| *Abl1* | -1.23 | 0.00999 | Abelson tyrosine-protein kinase 1 | Proto-oncogene C-ABL | Plays a role in many key processes linked to cell growth and survival such as cytoskeleton remodeling, cell motility and adhesion, receptor endocytosis, autophagy, DNA damage response, and apoptosis; regulates T cell differentiation by phosphorylating TBX21, leading to its enhancement |
| *Bid* | 1.2 | 0.00909 | BH3 interacting domain death agonist | Desmocollin type 4, apoptic death agonist | Induces caspases and apoptosis; counters the protective effect of BCL2, allowing release of cytochrome C |
| *Fas* | -0.893 | 0.0218 | Fragment apoptosis stimulating |  | Cell surface death receptor; interaction with FAS-ligand triggers an apoptotic signaling cascade; also activates NFκB, ERK1, and MAPK8 |
| *Ifitm2* | -0.971 | 0.0275 | Interferon-induced transmembrane protein 2 |  | IFN-induced antiviral protein which inhibits the entry of viruses to the host cell cytoplasm; induces cell cycle arrest and mediates p53-independent apoptosis through caspase activation |
| *Trp53* | -0.761 | 0.0025 | Transformation-related protein 53 |  | TF that induces cell-cycle arrest and apoptosis through stimulation of Fas expression |
| ***Angiogenesis*** | | | | | |
| *Cd97* | -1.23 | 0.00591 | Cluster of differentiation 97 | BL-Ac[F2] | GPCR that promotes granulocyte adhesion and migration; activates T cells *via* binding to CD55; stimulates angiogenesis through binding integrin counterreceptors on endothelial cells |
| ***Antigen Processing & Presentation*** | | | | | |
| *Cd1d1* | 0.852 | 0.0131 | Antigen-presenting glycoprotein CD1d1 |  | Murine non-classical class I MHC; primarily presents lipid and glycolipid Ags |
| *Cd1d2* | 1.38 | 0.00326 | Antigen-presenting glycoprotein CD1d2 |  | Pairs with CD1d1 to form the murine non-classical class I MHC, CD1d; primarily presents lipid and glycolipid Ags; essential for NKT cell development; presents shorter acyl chain Ags than CD1d1 |
| *Cd83* | -1.45 | 0.0391 | Cluster of differentiation 83 |  | APC surface marker; may be involved in the regulation of Ag presentation |
| *Icam1* | -1.29 | 0.0226 | Intracellular adhesion molecule 1 |  | Cell surface glycoprotein that serves as strong adhesive ligand for LFA-1; important for leukocyte mobility and costimulation |
| *H60a* | -1.41 | 0.0467 | Histocompatibility 60a |  | Enables NK cell lectin-like receptor binding activity |
| ***Autophagy*** | | | | | |
| *Abl1* | -1.23 | 0.00999 | Abelson tyrosine-protein kinase 1 | Proto-oncogene C-ABL | Plays a role in many key processes linked to cell growth and survival such as cytoskeleton remodeling, cell motility and adhesion, receptor endocytosis, autophagy, DNA damage response, and apoptosis; regulates T cell differentiation by phosphorylating TBX21, leading to its enhancement |
| *Atg12* | -0.785 | 0.0153 | Autophagy related 12 |  | Pairs with ATG5 to promote the extension of the phagophoric membrane in autophagic vesicles |
| ***B Cell-associated Genes*** | | | | | |
| *Blnk* | -1.72 | 0.0354 | B cell linker | Src homology 1 domain-containing leukocyte protein of 65 kDa (SLP-65); Ly57 | Functions as a central linker protein downstream of the B cell receptor, bridging SYK kinase to a multitude of signaling pathways and regulating biological outcomes of B cell function and development; plays a role in the activation of ERK/EPHB2, MAP kinase p38 and JNK; modulates AP1 activation; important for the activation of NFκB and NFAT |
| *Cd19* | -2.87 | 0.0306 | Cluster of differentiation 19 | B-lymphocyte surface antigen B4 | BCR coreceptor; activates signaling pathways that lead to the activation of PI3K and Ca2+ flux |
| *Cd79b* | -2.44 | 0.0421 | Cluster of differentiation 79b | B29 | One of the two flanking proteins that initiate signaling downstream of the BCR |
| *Cd81* | -1.7 | 0.0133 | Cluster of differentiation 81 | Tetraspanin-28 | Acts as a platform for receptor clustering and signaling; essential for trafficking and compartmentalization of CD19 receptor on the surface of activated B cells; facilitates the localization of CD3ζ at antigen-induced synapses with B cells; may also play a role in antigen presentation |
| *Fcgr4* | 2.22 | 0.0295 | Fragment crystallizable gamma receptor 4 | Fc receptor-like 3 (Fcrl3); CD16-2 | Putative mouse ortholog to human FcγRIIIA |
| *Icosl* | -1.34 | 0.00101 | Inducible T cell costimulator ligand | CD275 | Ligand for T cell-specific co-receptor ICOS; also induces B cell proliferation and plasma cell differentiation |
| ***Calcium Signaling*** | | | | | |
| *S100b* | -2.93 | 0.00372 | S100 Ca2+ binding protein B |  | Weakly binds Ca2+ but binds zinc very tightly; may mediate Ca2+-dependent regulation of many physiological processes by interacting with other proteins, such as TPR-containing proteins, and modulating their activity |
| ***Chemotaxis*** | | | | | |
| *Ccl1* | 1.31 | 0.0367 | C-C motif chemokine ligand 1 | T lymphocyte-secreted protein I-309; small inducible cytokine A1 | Chemoattractant for monocytes but not neutrophils; binds to CCR8 |
| *Ccl11* | -1.58 | 0.00536 | C-C motif chemokine ligand 11 | Eotaxin | Chemoattractant for eosinophils |
| *Ccl17* | 2.28 | 0.0238 | C-C motif chemokine ligand 17 |  | Chemoattractant ligand for CCR4 and -8; attracts T cells |
| *Ccl3* | 2.73 | 0.0185 |  | C-C motif chemokine ligand 3 | Macrophage inflammatory protein 1α (MIP1α) |
| *Ccl7* | 1.29 | 0.0196 | C-C motif chemokine ligand 7 | Monocte chemotactic protein 3 (MCP3) | General chemokine that recruits leukocytes to infected tissues; mainly observed in monocyte mobilization |
| *Ccnd3* | -1.13 | 0.00455 | Cyclin D3 |  | Regulatory component of the cyclin D3-CDK4 complex that inhibitively phosphorylates members of the retinoblastoma protein family; regulates the cell-cycle during G(1)/S transition |
| *Ccr1* | 2.07 | 0.0137 | C-C motif chemokine receptor 1 | MIP1α receptor | Receptor for CCL3, -5, -7, and -23 |
| *Ccr7* | -2.51 | 0.00929 | C-C chemokine receptor type 7 | CD197 | Chemokine receptor that activates B and T cells and promotes their homing to secondary lymphoid organs; also stimulates DC expression of MHC class I and II |
| *Ccr8* | -1.57 | 0.044 | C-C motif chemokine receptor 8 |  | Receptor for CCL1; may regulate monocyte chemotaxis and thymic cell line apoptosis |
| *Cxcl11* | 2.17 | 0.0148 | C-X-C motif chemokine ligand 11 |  | Dominant ligand for CXCR3; attracts activated T cells; strongly induced by IFNγ |
| *Cxcl2* | 2.62 | 0.0042 | C-X-C motif chemokine ligand 2 | Macrophage inflammatory protein 2-alpha (MIP2α); GRO2 oncogene | Chemokine produced by activated monocytes and neutrophils and expressed at sites of inflammation |
| *Cxcl3* | 7.72 | 0.000688 | C-X-C motif chemokine ligand 3 | GRO3 oncogene | Ligand for CXCR2; attracts neutrophils |
| *Cxcl9* | 1.47 | 0.0449 | C-X-C motif chemokine ligand 9 | Humig | Chemoattractant ligand for CXCR3; attracts activated T cells |
| *Cxcr4* | -1.75 | 0.00672 | C-X-C motif chemokine receptor 4 | Fusin; CD184 | Alpha-chemokine receptor specific for SDF1 *aka* CXCL12 |
| *Cxcr5* | -3.4 | 0.0272 | C-X-C motif chemokine receptor 5 | CD185; Burkitt's lymphoma receptor 1 (BLR1) | Cytokine receptor that binds to B lymphocyte chemoattractant (BLC); involved in B cell migration into splenic follicles and Peyer's patches |
| ***Complement & Humoral Immunity*** | | | | | |
| *C6* | 1.73 | 0.00661 | Complement component 6 |  | Part of the membrane attack complex |
| *Cfb* | 1.4 | 0.0327 | Complement factor B |  | Alternate complement pathway component; when cleaved, produces a serine protease that binds to C3b to form C3 convertase |
| *Fcgr4* | 2.22 | 0.0295 | Fragment crystallizable gamma receptor 4 | Fc receptor-like 3 (Fcrl3); CD16-2 | Putative mouse ortholog to human FcγRIIIA |
| ***Co-stimulation*** | | | | | |
| *Icosl* | -1.34 | 0.00101 | Inducible T cell costimulator ligand | CD275 | Ligand for T cell-specific co-receptor ICOS; also induces B cell proliferation and plasma cell differentiation |
| ***Cytokines*** | | | | | |
| *Il1a* | 3.38 | 0.0226 | Interleukin 1 alpha | Hematopoietin-1 | Cytokine produced by monocytes and macrophages in response to cell injury; stimulates thymocyte proliferation by inducing IL-2 release; also stimulates B cell maturation and proliferation, and fibroblast growth factor activity |
| *Csf2* | 3.12 | 0.00602 | Colony-stimulating factor 2 | Sargramostim | Cytokine that stimulates the growth and differentiation of hematopoietic precursor cells from various lineages, including granulocytes, macrophages, eosinophils, and erythrocytes |
| *Il1b* | 2.87 | 0.00578 | Interleukin 1 beta | Catabolin | One of the two primary inflammatory cytokines produced by the inflammasome (the other one being IL-18); induces neutrophil influx and activation,T cell activation and cytokine production, B-cell activation and antibody production, fibroblast proliferation, and collagen production; synergizes with IL-12 to induce IFNγ synthesis from Th1 cells |
| *Spp1* | 2.58 | 0.00606 | Secreted phosphoprotein 1 | Osteopontin | Cytokine involved in enhancing production of IFNγ and IL-12 and reducing production of IL-10 |
| *Il12rb1* | 2.56 | 0.0247 | Interleukin 12 receptor subunit beta 1 | CD212 | Cytokine receptor component that associates with IL12RB2 to IL23R |
| *Il1r2* | 1.46 | 0.0166 | Interleukin 1 receptor type II | CD121b | Non-signaling receptor for IL-1α, -β, and RN; serves as a decoy receptor by competetive binding to IL-1β and preventing its binding to IL1R1 |
| *Il4ra* | -0.609 | 0.0435 | Interleukin 4 receptor subunit alpha | CD124 | Alpha chain for the IL-4 and IL-13 receptors; involved in Th2 differentiation and IgE production |
| *Il15* | -0.621 | 0.0362 | Interleukin 15 |  | Stimulates T cell proliferation and phagocytosis in neutrophils |
| *Ifnar1* | -0.651 | 0.0151 | Interferon-alpha/beta receptor alpha chain |  | Component of the receptor for type I IFNs, binding of which activates the JAK-STAT pathway |
| *Ifngr1* | -0.991 | 0.00506 | Interferon gamma receptor 1 | CD54 | One of the two components of the IFNγ receptor; stimulates activation of the JAK/STAT signaling pathway |
| *Il6st* | -1.82 | 0.0103 | Interleukin 6 cytokine family signal transducer | Glycoprotein 130 (Gp130); CD130 | Transmembrane protein that acts a component in several cytokine receptors, including IL-6 |
| *Tgfb3* | -1.84 | 0.0276 | Transforming growth factor beta 3 |  | Multifunctional protein that regulates embryogenesis and cell differentiation |
| *Il34* | -1.98 | 0.0088 | Interleukin 34 |  | Cytokine that promotes the proliferation, survival and differentiation of monocytes and macrophages, as well as the release of proinflammatory chemokines |
| *Ifna1* | -2.01 | 0.0201 | Interferon alpha 1 |  | Macrophage-produced antiviral, pro-inflammatory cytokine |
| *Il6* | -2.44 | 0.00404 | Interleukin 6 |  | Pro-inflammatory cytokine that signals through the JAK and STAT pathways |
| ***Inflammation*** | | | | | |
| *Ifna1* | -2.01 | 0.0201 | Interferon alpha 1 |  | Macrophage-produced antiviral, pro-inflammatory cytokine |
| *Ifnar1* | -0.651 | 0.0151 | Interferon-alpha/beta receptor alpha chain |  | Component of the receptor for type I IFNs, binding of which activates the JAK-STAT pathway |
| *Ifngr1* | -0.991 | 0.00506 | Interferon gamma receptor 1 | CD54 | One of the two components of the IFNγ receptor; stimulates activation of the JAK/STAT signaling pathway |
| *Il1a* | 3.38 | 0.0226 | Interleukin 1 alpha | Hematopoietin-1 | Cytokine produced by monocytes and macrophages in response to cell injury; stimulates thymocyte proliferation by inducing IL-2 release; also stimulates B cell maturation and proliferation, and fibroblast growth factor activity |
| *Il1b* | 2.87 | 0.00578 | Interleukin 1 beta | Catabolin | One of the two primary inflammatory cytokines produced by the inflammasome (the other one being IL-18); induces neutrophil influx and activation,T cell activation and cytokine production, B-cell activation and antibody production, fibroblast proliferation, and collagen production; synergizes with IL-12 to induce IFNγ synthesis from Th1 cells |
| *Il6* | -2.44 | 0.00404 | Interleukin 6 |  | Pro-inflammatory cytokine that signals through the JAK and STAT pathways |
| *Il6st* | -1.82 | 0.0103 | Interleukin 6 cytokine family signal transducer | Glycoprotein 130 (Gp130); CD130 | Transmembrane protein that acts a component in several cytokine receptors, including IL-6 |
| *Irf1* | -1.46 | 0.00796 | Interferon regulatory factor 1 |  | Transcriptional regulator that promotes inflammatory innate and adaptive immune responses |
| *Irf3* | -0.894 | 0.0268 | Interferon regulatory factor 3 |  | Complexes with CREBBP to translocate to the nucleus and transcriptionally activate type I IFNs |
| *Irf4* | -1.58 | 0.005 | Interferon regulatory factor 4 |  | Transcriptional activator that complexes with BATF and binds ISREs within the promoters of multiple genes involved in inflammation |
| *Isg15* | -1.96 | 0.00941 | Interferon-stimulated gene 15 |  | Ubiquitin-like protein that binds intracellular target proteins upon activation by IFNα or β; can also be secreted to induce NK cell proliferation, act as a chemoattractant for neutrophils, and induce IFNγ upon binding to ITGAL/ITGB2 |
| *Mx2* | -3.14 | 0.000635 | Myxovirus resistance protein 2 |  | IFN-induced dynamin-like GTPase with potent antiviral activity against HIV-1 |
| *Pin1* | -1.84 | 0.00234 | Peptidylprolyl cis/trans isomerase, NIMA-interacting 1 |  | Acts as a molecular switch in multiple cellular processes; inhibits mitosis presumably by interacting with NIMA and attenuating its mitosis-promoting activity; downregulates kinase activity of BTK; induces IRAK3 stabilization, nuclear translocation, and expression of pro-inflammatory genes in dendritic cells |
| *Spp1* | 2.58 | 0.00606 | Secreted phosphoprotein 1 | Osteopontin | Cytokine involved in enhancing production of IFNγ and IL-12 and reducing production of IL-10 |
| *Tbk1* | -0.829 | 0.0235 | TANK-binding kinase 1 |  | Coordinates the activation of IRF3 and NFκB and induction of type I IFNs |
| ***Inhibition*** | | | | | |
| *Bcl2* | -0.946 | 0.0163 | B cell lymphoma 2 |  | Outer mitochondrial membrane protein that inhibits apoptosis and autophagy; may attenuate inflammation by impairing inflammasome formation |
| *Bcl2l1* | -1.22 | 0.0333 | B cell lymphoma 2 like 1 | Protein phosphatase 1 | Potent inhibitor of caspase-mediated cell death |
| *Btla* | -2.46 | 0.000221 | B and T lymphocyte attenuator | CD272 | Inhibitory cell surface protein that inhibits T cell function by binding to B7H4 and TNFRSF14 |
| *Chuk* | -0.722 | 0.00208 | Conserved helix-loop-helix ubiquitous kinase | Inhibitor of NFκB kinase subunit alpha (IKKα) | Part of the IKK complex that inhibits IκBα and permits NFκB nuclear localization |
| *Cyld* | -0.68 | 0.0301 | Cylindromatosis lysine 63 deubiquitinase |  | Inhibits NFκB activation by deubiquitinating upstream signaling factors; inhibits Wnt signaling; restricts polyubiquitination of RIPK1 and -2, thereby limiting necroptosis |
| *Cd28* | -1.36 | 0.0473 | Cluster of differentiation 28 |  | Essential T cell co-receptor that enhances T cell activation, proliferation, cytokine production, and survival; binds to CD80 and CD86 |
| *Cdkn1a* | -1.16 | 0.0286 | Cyclin dependent kinase inhibitor 1A | p21; CDK-interaction protein 1 (CIP1) | Binds to and inhibits cyclin-dependent kinase activity, preventing phosphorylation of critical cyclin-dependent kinase substrates and blocking cell cycle progression |
| *Dusp4* | -1.3 | 0.0156 | Dual specificity phosphatase 4 |  | Inactivates ERK1, ERK2, and JNK |
| *Ikbkb* | -0.715 | 0.0106 | Inhibitor of nuclear factor kappa B kinase subunit beta |  | Part of the IKK complex that inhibits IκBα and permits NFκB nuclear localization |
| *Ikbkg* | -0.692 | 0.0168 | Inhibitor of nuclear factor kappa B kinase subunit gamma | NFκB essential modifier (NEMO) | Regulatory subunit of the IKK core complex that phosphorylates inhibitors of NFκB thus leading to the dissociation of the inhibitor/NFκB complex and ultimately the degradation of the inhibitor |
| *Klra2* | 2.67 | 0.0168 | Killer cell lectin-like receptor subfamily A member 2 | Ly49b | Inhibitor receptor on NK cells for MHC class I; recruits SHP1, -2, and SHIP phosphatases upon binding to its ligand |
| *Nfkbia* | -0.911 | 0.0239 | Nuclear factor kappa B inhibitor alpha |  | Inhibits activity of REL dimers by masking of their nuclear localization signals |
| *Pin1* | -1.84 | 0.00234 | Peptidylprolyl cis/trans isomerase, NIMA-interacting 1 |  | Acts as a molecular switch in multiple cellular processes; inhibits mitosis presumably by interacting with NIMA and attenuating its mitosis-promoting activity; downregulates kinase activity of BTK; induces IRAK3 stabilization, nuclear translocation, and expression of pro-inflammatory genes in dendritic cells |
| *Pvrl2* | -0.693 | 0.0455 | Poliovirus receptor-related protein 2 | Nectin-2 | Variable costimulator/coinhibitor of T cell function, depending on which receptor it binds to: stimulates T cell proliferation and cytokine production upon binding to CD226; inhibits T cell proliferation upon interaction with PVRIG |
| *Sigirr* | -1.31 | 0.00408 | Single Ig domain-containing IL-1R-related protein |  | Negative regulator of the Toll-like and IL-1R receptor signaling pathways; attenuates the recruitment of receptor-proximal signaling components to the TLR4 receptor; interferes with the heterodimerization of Il1R1 and IL1RAP |
| *Socs3* | -1.45 | 0.0171 | Suppressor of cytokine signaling 3 |  | Inhibits IL6ST and JAK2; negative regulator of IL-6 |
| ***IRAKs & TRAFs*** | | | | | |
| *Traf3* | -0.605 | 0.0128 | Tumor necrosis factor receptor-associated factor 3 |  | Adaptor protein that acts in the CD40 signaling cascade; induces NFκB and MAPK activation |
| ***JAK-STAT Pathway*** | | | | | |
| *Jak1* | -0.811 | 0.0331 | Janus kinase 1 |  | Essential tyrosine kinase involved signal transduction in type I and II cytokines and IFNs |
| *Jak2* | -1.2 | 0.00328 | Janus kinase 2 |  | Tyrosine kinase that participates in IFN and IL6ST signaling cascades |
| *Jak3* | -0.839 | 0.0155 | Janus kinase 3 |  | Non-receptor tyrosine kinase involved in various processes such as cell growth, development, or differentiation; mediates essential signaling events in both innate and adaptive immunity |
| *Stat1* | 0.824 | 0.0317 | Signal transducer and activator of transcription 1 |  | Transcriptional activator that mediates cellular responses to IFNs, cytokines, and other growth factors |
| *Stat3* | -1.03 | 0.012 | Signal transducer and activator of transcription 3 |  | Transcriptional activator of genes involved in cell growth and apoptosis; activated by JAKs |
| *Stat4* | -0.94 | 0.0107 | Signal transducer and activator of transcription 4 |  | Essential TF for Th1 CD4+ T cell development and IFNγ production; also promotes expression of MyD88 |
| ***MAP Kinase Signaling*** | | | | | |
| *Map3k1* | -1.39 | 0.000125 | Mitogen-activated protein kinase kinase kinase 1 |  | Serine/threonine kinase that activates the ERK and JNK kinase pathways by phosphorylation of MAP2K1 and MAP2K4; also activates CHUK and IKBKB, the central protein kinases of the NFκB pathway |
| *Map3k5* | -1.44 | 0.00264 | Mitogen-activated protein kinase kinase kinase 5 | Apoptosis signal-regulating kinase 1 | Essential component of the MAP kinase signal transduction pathway; mediates signaling for determination of cell fate such as differentiation and survival; plays a crucial role in the apoptosis signal transduction pathway through mitochondria-dependent caspase activation; required for the innate immune response; mediates signal transduction of receptor-mediated inflammatory signals, such as TNF or LPS |
| *Map3k7* | -0.743 | 0.0428 | Mitogen-activated protein kinase kinase kinase 7 | TGFβ-activated kinase (TAK1) | Signal transducer downstream of TGFβ and BMP; controls a variety of cell functions, including transcription regulation and apoptosis |
| *Map4k2* | -2.22 | 0.000542 | Mitogen-activated protein kinase kinase kinase kinase 2 |  | Essential component of the MAP kinase signal transduction pathway downstream of TRAF6; upstream activator of the SAP/JNK signaling pathway |
| *Mapk1* | -0.537 | 0.0472 | Mitogen-activated protein kinase 1 | Extracellular signal-regulated kinase 2 (ERK2) | Serine/threonine kinase that acts as an essential component of the MAP kinase signal transduction pathway |
| *Mapk3* | -1.47 | 0.00233 | Mitogen-activated protein kinase 3 | Extracellular signal-regulated kinase 1 (ERK1) | Serine/threonine kinase that acts as an essential component of the MAP kinase signal transduction pathway |
| *Mapk8* | -0.848 | 0.0423 | Mitogen-activated protein kinase 8 | c-Jun N-terminal kinase 1 (JNK1); Stress-activated protein kinase 1c (SAPK1) | Serine/threonine-protein kinase involved in various processes such as cell proliferation, differentiation, migration, transformation and programmed cell death; phosphorylates a number of transcription factors, primarily components of AP-1 such as JUN, JDP2, and ATF2, thus regulating AP-1 transcriptional activity; promotes stressed cell apoptosis by phosphorylating key regulatory factors including p53/TP53 and Yes-associates protein YAP1; required for Th1 differentiation |
| ***Metabolism*** | | | | | |
| *Ada* | -0.562 | 0.00542 | Adenosine deaminase |  | Key enzyme in purine metabolism; primarily involved in the development and maintenance of the immune system in humans |
| *Cd36* | -2.49 | 0.00409 | Cluster of differentiation 36 | Fatty acid translocase (FAT) | Class B scavenger receptor that mediates fatty acid uptake |
| *Pparg* | -2.43 | 0.000677 | Peroxisome proliferator activated receptor gamma |  | Nuclear receptor that binds peroxisome proliferators such as hypolipidemic drugs and fatty acids; once activated binds to specific PPAR response elements (PPRE) and modulates the transcription of its target genes, such as acyl-CoA oxidase, thereby controlling the peroxisomal beta-oxidation pathway of fatty acids |
| ***NFκB Signaling*** | | | | |  |
| *Bcl10* | -0.682 | 0.0354 | B cell lymphoma/leukemia 10 |  | Activates NFκB *via* ubiquitination of IKKγ |
| *Nfkb1* | -0.834 | 0.0065 | Nuclear factor kappa B subunit 1 | p105/p50 | One of the NFκB family TFs; inhibits inflammation |
| *Rel* | -1.65 | 0.000363 | Avian reticuloendotheliosis viral oncogene homolog | c-Rel | One of the NFκB family TFs; important for B cell and Treg development |
| *Rela* | -1.16 | 0.00571 | Avian reticuloendotheliosis viral oncogene homolog A | p65 | One of the NFκB family TFs; major driver of inflammation |
| *Relb* | -0.537 | 0.0306 | Avian reticuloendotheliosis viral oncogene homolog B |  | One of the NFκB family TFs; controls lymphoid development, DC biology, and noncanonical NFκB signaling |
| *Ripk2* | -1 | 0.0212 | Receptor-interacting serine/threonine-protein kinase 2 |  | RIP kinase that potentiates signals downstream of NOD1 and -2, leading to NFκB activation; promotes BCL10 phosphorylation and subsequent NFκB activation following TCR engagement |
| ***NK Cell Function*** | | | | | |
| *Gzmb* | 2.53 | 0.0166 | Granzyme B |  | Abundant protease in the cytosolic granules of cytotoxic T and NK cells that activates caspase-mediated cell death when delivered into the target cell through the immunological synapse |
| *Pvr* | -1.53 | 0.006 | Poliovirus receptor | CD155 | Mediates NK cell adhesion and triggers NK cell effector functions; binds CD96 and CD226, leading to the formation of a mature immunological synapse between NK cell and target cell |
| *Tcf7* | -1.81 | 0.0123 | Transcription factor 7 |  | HMG box TF predominantly expressed by T cells that drives their development, although also involved in NK cell development; activates transcription through a Wnt/β-catenin signaling pathway |
| ***Pattern Recognition Receptors*** | | | | | |
| *Clec4n* | 2.74 | 0.0278 | C-type lectin domain family 4, member N | Dectin-2 | PRR specific for Mycobacterial mannose-capped lipoarabinomannan |
| *Clec5a* | 2.55 | 0.048 | C-Type lectin domain family 5, member A | Myeloid DAP12-associating lectin-1 | Critical macrophage receptor for dengue virus serotypes 1-4; positive regulator of osteoclastogenesis |
| *Ddx58* | -1.05 | 0.0111 | DExD/H-box helicase 58 | Retinoic acid-inducible gene I (RIG-I) | Cytoplasmic PRR that recognizes dsRNA; can promote T cell-independent B cell activation; uses MAVS as an adaptor |
| *Fpr2* | 2.83 | 0.0126 | Formyl peptide receptor 2 | Lipoxin A4 receptor | Low affinity receptor for N-formyl-methionyl peptides; activates neutrophils |
| *Marco* | 2.64 | 0.012 | Macrophage receptor with collagenous structure |  | A PRR that recognizes LDL |
| *Myd88* | -1.55 | 0.0156 | Myeloid differentiation primary response 88 |  | Key adaptor in the TLR signaling pathways; interacts with all TLRs except TLR3; activates NFκB and IRFs |
| *Nod1* | -0.994 | 0.0221 | Nucleotide binding oligomerization domain containing 1 |  | Intracellular PRR that recognizes peptidoglycan-derived muropeptides and *Shigella* effector proteins |
| *Tlr3* | -1.37 | 0.0164 | Toll-like receptor 3 | CD283 | Endosomic PRR that recognizes dsRNA |
| *Tollip* | -1.05 | 0.00843 | Toll interacting protein |  | Inhibitory adaptor protein that acts upon TLR2 |
| ***ROS Generation*** | | | | | |
| *Nos2* | 1.81 | 0.0136 | Inducible nitric oxide synthase (iNOS) |  | Produces reactive oxygen species and contributes to inflammatory cytokine production |
| *Pparg* | -2.43 | 0.000677 | Peroxisome proliferator activated receptor gamma |  | Nuclear receptor that binds peroxisome proliferators such as hypolipidemic drugs and fatty acids; once activated binds to specific PPAR response elements (PPRE) and modulates the transcription of its target genes, such as acyl-CoA oxidase, thereby controlling the peroxisomal beta-oxidation pathway of fatty acids |
| *Txnip* | -1.05 | 0.0426 | Thioredoxin interacting protein |  | Thiol-oxidoreductase; protects cells from oxidative stress by inhibiting thioredoxin |
| ***T Cell Function*** | | | | | |
| *Abl1* | -1.23 | 0.00999 | Abelson tyrosine-protein kinase 1 | Proto-oncogene C-ABL | Plays a role in many key processes linked to cell growth and survival such as cytoskeleton remodeling, cell motility and adhesion, receptor endocytosis, autophagy, DNA damage response, and apoptosis; regulates T cell differentiation by phosphorylating TBX21, leading to its enhancement |
| *Cd97* | -1.23 | 0.00591 | Cluster of differentiation 97 | BL-Ac[F2] | GPCR that promotes granulocyte adhesion and migration; activates T cells *via* binding to CD55; stimulates angiogenesis through binding integrin counterreceptors on endothelial cells |
| *Gata3* | -1.62 | 0.00209 | GATA binding protein 3 |  | Transcriptional activator that binds to the enhancer of the TCR α and δ genes; required for Th2 differentiation following immune and inflammatory responses |
| *Gzmb* | 2.53 | 0.0166 | Granzyme B |  | Abundant protease in the cytosolic granules of cytotoxic T and NK cells that activates caspase-mediated cell death when delivered into the target cell through the immunological synapse |
| *Icosl* | -1.34 | 0.00101 | Inducible T cell costimulator ligand | CD275 | Ligand for T cell-specific co-receptor ICOS; also induces B cell proliferation and plasma cell differentiation |
| *Nfatc1* | -1.37 | 0.00394 | Nuclear factor of activated T cells, cytoplasmic 1 |  | Inducible nuclear component of the NFAT TF complex; mediates induction of IL-2 and IL-4 in T cells |
| *Nfatc2* | -1.03 | 0.0156 | Nuclear factor of activated T cells, cytoplasmic 2 |  | Cytosolic component of the NFAT TF complex; mediates induction of IL-2, IL-3, IL-4, TNFα, and GM-CSF |
| *Pvrl2* | -0.693 | 0.0455 | Poliovirus receptor-related protein 2 | Nectin-2 | Variable costimulator/coinhibitor of T cell function, depending on which receptor it binds to: stimulates T cell proliferation and cytokine production upon binding to CD226; inhibits T cell proliferation upon interaction with PVRIG |
| *Rora* | -1.19 | 0.0294 | Retinoic acid receptor-related orphan receptor A |  | Nuclear receptor that binds hormone response elements upstream of several genes to enhance the expression of those genes |
| *Tcf7* | -1.81 | 0.0123 | Transcription factor 7 |  | HMG box TF predominantly expressed by T cells that drives their development, although also involved in NK cell development; activates transcription through a Wnt/β-catenin signaling pathway |
| *Txk* | -1.23 | 0.0397 | TXK tyrosine kinase |  | Regulates the development, function, and differentiation of conventional T cells and nonconventional NK-T cells; contributes to signaling from many receptors and participates in multiple downstream pathways, including regulation of the actin cytoskeleton; can phosphorylate PLCγ1, leading to its localization in lipid rafts and activation, followed by subsequent cleavage of its substrates |
| ***Transcription Factors & Chromatin Remodellers*** | | | | | |
| *Atf2* | -1.04 | 0.00375 | Activating transcription factor 2 | Cyclic AMP-responsive element-binding protein 2 (CREB2) | Regulates transcription of various genes involved in anti-apoptosis, cell growth, and DNA damage response; in the nucleus, contributes to global transcription and the DNA damage response, in addition to specific transcriptional activities that are related to cell development, proliferation and death; in the cytoplasm, impairs mitochondrial membrane potential, inducing mitochondrial leakage and promoting cell death; phosphorylated form (mediated by ATM) plays a role in the DNA damage response |
| *Creb1* | -1.07 | 0.00431 | CAMP responsive element binding protein 1 |  | Phosphorylation-dependent transcription factor that stimulates transcription upon binding to the DNA cAMP response element (CRE), which is found in the promoter regions of several immune-related genes, including IL-2, IL-6, IL-10, and TNF-α; regulates diverse cellular responses, including proliferation, survival, and differentiation |
| *Crebbp* | -1.17 | 0.00584 | CREB binding protein | KAT3A | Binds specifically to phosphorylated CREB and enhances its transcriptional activity toward cAMP-responsive genes; also acetylates histones, giving a specific tag for transcriptional activation |
| *Egr1* | -1.85 | 0.00937 | Early growth response 1 | Zinc finger protein 268 (ZNF286) | Transcriptional repressor of genes involved in differentiations and mitogenesis; activates expression of p53 |
| *Ep300* | -0.887 | 0.0169 | Adenovirus early region 1A-associated protein p300 |  | Histone acetyltransferase; participates in chromatin remodeling to facilitate gene accessibility |
| *Gata3* | -1.62 | 0.00209 | GATA binding protein 3 |  | Transcriptional activator that binds to the enhancer of the TCR α and δ genes; required for Th2 differentiation following immune and inflammatory responses |
| *Hmgb1* | -0.961 | 0.0214 | High-mobility group box 1 |  | Remodels chromatin to make DNA more available for transcription |
| *Irf1* | -1.46 | 0.00796 | Interferon regulatory factor 1 |  | Transcriptional regulator that promotes inflammatory innate and adaptive immune responses |
| *Irf3* | -0.894 | 0.0268 | Interferon regulatory factor 3 |  | Complexes with CREBBP to translocate to the nucleus and transcriptionally activate type I IFNs |
| *Irf4* | -1.58 | 0.005 | Interferon regulatory factor 4 |  | Transcriptional activator that complexes with BATF and binds ISREs within the promoters of multiple genes involved in inflammation |
| *Nfkb1* | -0.834 | 0.0065 | Nuclear factor kappa B subunit 1 | p105/p50 | One of the NFκB family TFs; inhibits inflammation |
| *Pparg* | -2.43 | 0.000677 | Peroxisome proliferator activated receptor gamma |  | Nuclear receptor that binds peroxisome proliferators such as hypolipidemic drugs and fatty acids; once activated binds to specific PPAR response elements (PPRE) and modulates the transcription of its target genes, such as acyl-CoA oxidase, thereby controlling the peroxisomal beta-oxidation pathway of fatty acids |
| *Rel* | -1.65 | 0.000363 | Avian reticuloendotheliosis viral oncogene homolog | c-Rel | One of the NFκB family TFs; important for B cell and Treg development |
| *Rela* | -1.16 | 0.00571 | Avian reticuloendotheliosis viral oncogene homolog A | p65 | One of the NFκB family TFs; major driver of inflammation |
| *Relb* | -0.537 | 0.0306 | Avian reticuloendotheliosis viral oncogene homolog B |  | One of the NFκB family TFs; controls lymphoid development, DC biology, and noncanonical NFκB signaling |
| *Tcf7* | -1.81 | 0.0123 | Transcription factor 7 |  | HMG box TF predominantly expressed by T cells that drives their development, although also involved in NK cell development; activates transcription through a Wnt/β-catenin signaling pathway |
| *Yy1* | -0.827 | 0.0068 | Yin yang 1 |  | Ubiquitous factor that serves as a transcriptional "switch", either promoting or repressing the transcription of numerous genes through the selective recruitment of either histone deacetylases or acetyltransferases; plays a fundamental role in diverse processes, such as differentiation, replication, and cellular proliferation |
| ***Tyrosine Kinases*** | | | | | |
| *Itk* | -1.08 | 0.0289 | Interleukin-2-inducible T cell kinase | LYK | Key actor in the TCR signaling cascade; phosphorylates PLCγ1, LAT, and LCP2 |
| *Jak1* | -0.811 | 0.0331 | Janus kinase 1 |  | Essential tyrosine kinase involved signal transduction in type I and II cytokines and IFNs |
| *Jak2* | -1.2 | 0.00328 | Janus kinase 2 |  | Tyrosine kinase that participates in IFN and IL6ST signaling cascades |
| *Jak3* | -0.839 | 0.0155 | Janus kinase 3 |  | Non-receptor tyrosine kinase involved in various processes such as cell growth, development, or differentiation; mediates essential signaling events in both innate and adaptive immunity |
| *Tbk1* | -0.829 | 0.0235 | TANK-binding kinase 1 |  | Coordinates the activation of IRF3 and NFκB and induction of type I IFNs |
| *Txk* | -1.23 | 0.0397 | TXK tyrosine kinase |  | Regulates the development, function, and differentiation of conventional T cells and nonconventional NK-T cells; contributes to signaling from many receptors and participates in multiple downstream pathways, including regulation of the actin cytoskeleton; can phosphorylate PLCγ1, leading to its localization in lipid rafts and activation, followed by subsequent cleavage of its substrates |
| *Tyk2* | -0.947 | 0.0201 | Tyrosine kinase 2 | JTK1 | Plays both structural and catalytic roles in numerous cytokines and interferons signaling; associates with cytokine and growth factor receptors and activate STAT family members including STAT1, STAT3, STAT4, or STAT6 |
| ***Ubiquitin Regulation*** | | | | |  |
| *Bcl10* | -0.682 | 0.0354 | B cell lymphoma/leukemia 10 |  | Activates NFκB *via* ubiquitination of IKKγ |
| *Cyld* | -0.68 | 0.0301 | Cylindromatosis lysine 63 deubiquitinase |  | Inhibits NFκB activation by deubiquitinating upstream signaling factors; inhibits Wnt signaling; restricts polyubiquitination of RIPK1 and -2, thereby limiting necroptosis |
| *Itch* | -0.872 | 0.00379 | Itchy E3 ubiquitin protein ligase |  | Participates with TNFAIP3 in a ubiquitin-editing complex that marks components of inflammatory signaling pathways such as JUNB and CXCR4 for degradation |
| *Ubc* | -1.49 | 0.013 | Polyubiquitin C |  | Serves various roles, including immate immunity, DNA repair, and stimulation of autophagy and the proteasomal response |
| ***Other*** | | | | | |
| *Atm* | -0.471 | 0.04 | Ataxia telangiectasia mutated |  | Serine/threonine protein kinase that activates checkpoint signaling upon DSBs, apoptosis, and genotoxic stresses; acts as a master controller for cell cycle checkpoint signaling pathways required for the DNA damage response and genomic stability |
| *Rps6* | -0.821 | 0.0119 | Ribosomal protein 6 |  | Component of the 40S small ribosomal subunit; plays an important role in controlling cell growth and proliferation through the selective translation of particular classes of mRNA |
